# Luminescent Iridium Complex-Peptide Hybrids (IPHs) for Therapeutics of Cancer: Design and Synthesis of IPHs for Detection of Cancer Cells and Induction of Their Necrosis-Type Cell Death

**DOI:** 10.1155/2018/7578965

**Published:** 2018-08-01

**Authors:** Abdullah-Al Masum, Yosuke Hisamatsu, Kenta Yokoi, Shin Aoki

**Affiliations:** ^1^Faculty of Pharmaceutical Sciences, Tokyo University of Science, 2641 Yamazaki, Noda, Chiba 278-8510, Japan; ^2^Imaging Frontier Center, Tokyo University of Science, 2641 Yamazaki, Noda, Chiba 278-8510, Japan

## Abstract

Death receptors (DR4 and DR5) offer attractive targets for cancer treatment because cancer cell death can be induced by apoptotic signal upon binding of death ligands such as tumor necrosis factor-related apoptosis-inducing ligand (TRAIL) with death receptors. Cyclometalated iridium(III) complexes such as *fac*-Ir(tpy)_3_ (tpy = 2-(4-tolyl)pyridine) possess a *C*_3_-symmetric structure like TRAIL and exhibit excellent luminescence properties. Therefore, cyclometalated Ir complexes functionalized with DR-binding peptide motifs would be potent TRAIL mimics to detect cancer cells and induce their cell death. In this study, we report on the design and synthesis of *C*_3_-symmetric and luminescent Ir complex-peptide hybrids (IPHs), which possess cyclic peptide that had been reported to bind DR5. The results of 27 MHz quartz-crystal microbalance (QCM) measurements of DR5 with IPHs and costaining experiments of IPHs and anti-DR5 antibody, suggest that IPHs bind with DR5 and undergo internalization into cytoplasm, possibly via endocytosis. It was also found that IPHs induce slow cell death of these cancer cells in a parallel manner to the DR5 expression level. These results indicate that IPHs may offer a promising tool as artificial luminescent mimics of death ligands to develop a new category of anticancer agents that detect and kill cancer cells.

## 1. Introduction

Death receptors (DRs) are often overexpressed on the cell membrane of cancer cells and bind with death ligands such as tumor necrosis factor-related apoptosis-inducing ligand (TRAIL) to send the cell extrinsic apoptotic signal [1]. TRAIL receptors comprise five categories, plasma membrane-expressed TRAIL-R1 (DR4), TRAIL-R2 (DR5), TRAIL-R3 (DcR1), TRAIL-R4 (DcR2), and a soluble receptor, osteoprotegerin (OPG). DR4 and DR5 contain death domain (DD) to transduce apoptotic signals and hence named as death receptors, whereas DcR1 and DcR2 are unable to induce cell death and regarded as decoy receptors [1–7]. TRAIL is a *C*_3_-symmetric protein, consists of three monomeric units, and binds with three DRs [8, 9]. Upon binding of DR with TRAIL, TRAIL-DR cluster sets up death-inducing signaling complex (DISC) at their cytoplasmic death domain (DD) and recruits adaptor protein (FADD) via death-effector domain (DED). The signaling activates procaspase-8 to caspase-8 and then caspase-3 in order to cleave multiple substrates to execute cell death [10]. The nonsignaling DcR1 does not contain cytoplasmic DD, and DcR2 is very similar to death receptors but contains a truncated form of DD. Therefore, both DcR1 and DcR2 are unable to assemble DISC [4–6].

Since death receptors are overexpressed in various types of cancer cells, TRAIL is capable of selectively inducing apoptosis of cancer cells with low cytotoxicity in normal cells [11–19]. Therefore, death receptors have been conceived as promising targets for the treatment and imaging of cancer cells. To date, only limited examples of artificial death receptor binders having TRAIL-like functionalities are reported. Representative examples include DR5 binding peptides [20–26], zinc-binding site peptide of TRAIL (RNSCWSKD that was screened out from TRAIL (227–234)) [27], and small molecular TRAIL mimics (bioymifi) [28].

**Figure 1:**
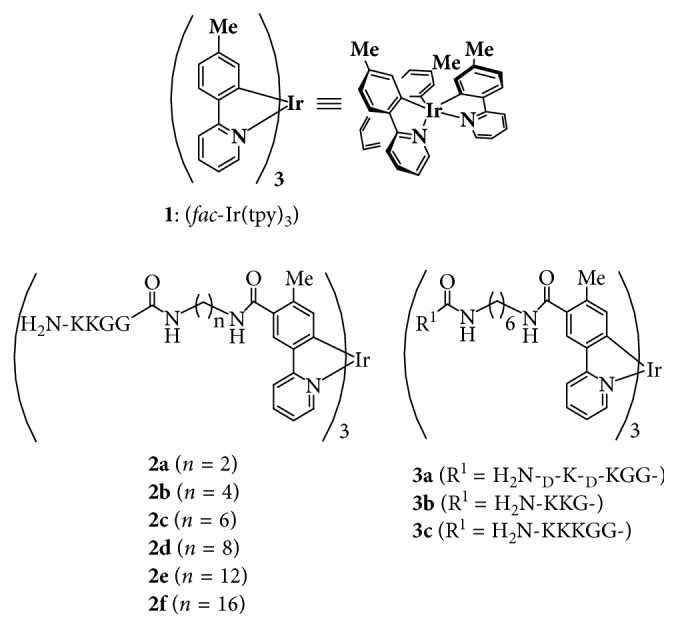
Ir complexes having cationic peptides.

Meanwhile, cyclometalated iridium (Ir(III)) complexes such as *fac*-Ir(tpy)_3_ **1** (tpy = 2-(4-tolyl)pyridine) (Figure 1) draw increasing attention as one of the imaging tools to study extra- and intracellular events, in addition to organic light-emitting diodes (OLEDs) such as phosphorescent emitters [29–33] because of their significant stability and excellent photophysical properties under physiological conditions [31, 33–39]. Such types of Ir complex analogues have been widely applied to oxygen sensors [40, 41], chemosensors [42–47], and luminescent probes for biological systems [48–66]. These advantages originate from their high-luminescence quantum yields, the long luminescence lifetimes (*τ*∼*µ*s) that can eliminate the short-lived autofluorescence (*τ*∼ns) from biological samples in cellular imaging, and significant stokes shift that minimizes self-quenching process [29–39]. We previously reported some examples of Ir complexes that can be functionalized as blue∼green∼red and white color emitters [67–71], pH sensors [68–70], photosensitizers [68–70], and cell death inducer of cancer cells [68–70]. Recently, we have reported on Ir complexes (**2a-f** and **3a-c**) having cationic peptides (typically, H_2_N-KKGG-) (Figure 1) as inducers and detectors of cell death of Jurkat cells (a human T-lymphoma cell line) [72–74]. These results suggest that Ir complexes are potential agents for the diagnosis and treatment of cancer and related diseases and even for mechanistic study of cell death processes.

**Figure 2:**
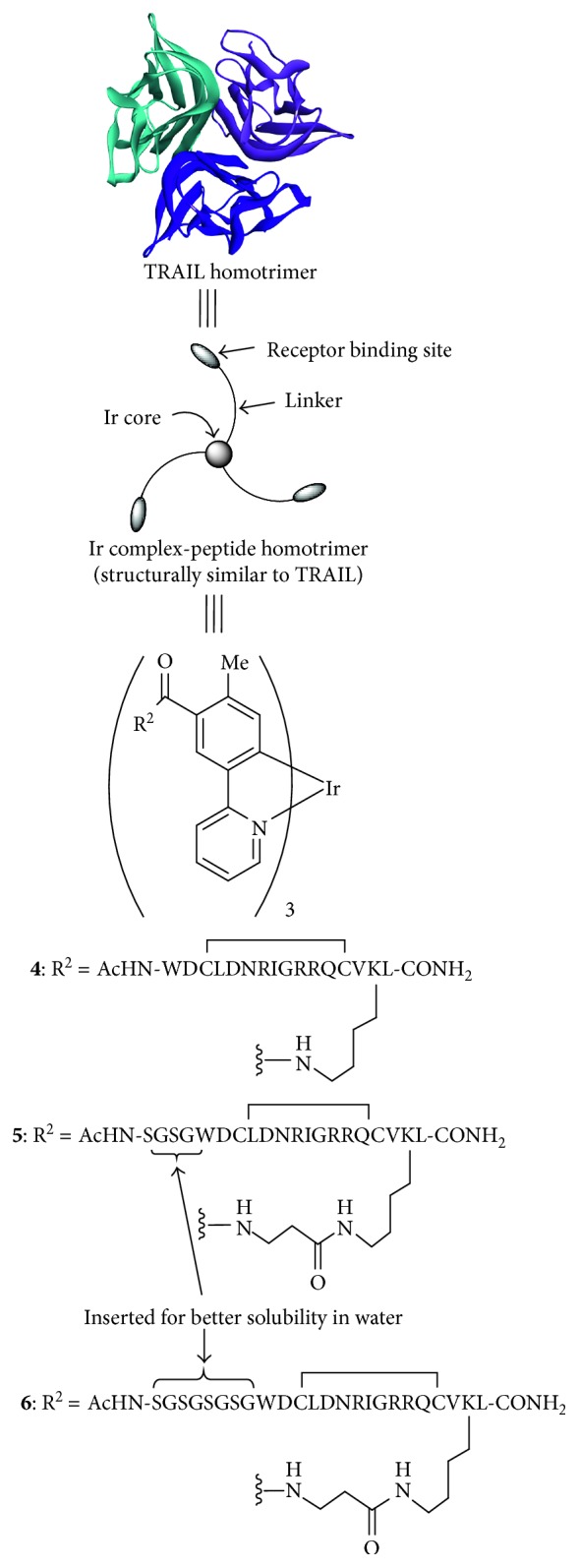
*C*_3_-symmetric tris-cyclometalated Ir complex-peptide hybrids (IPHs).

Because cyclometalated iridium (Ir(III)) complexes such as *fac*-Ir(tpy)_3_ **1** possess a *C*_3_-symmetric structure like TRAIL (the top of Figure 2), it is hypothesized that these complexes could be good scaffolds to mimic TRAIL. In this manuscript, we report on the design and synthesis of some new *C*_3_-symmetric tris-cyclometalated Ir complexes having cyclic peptides (Figure 2) [20–22], which had been reported to be able to bind DR5, for selective staining and induction of cell death of cancer cells. The Ir complexes **4**–**6** were synthesized by regioselective substitution reactions reported by us [75] and the successive coupling reactions with cyclic peptides [20–22]. Due to low solubility of **4** in water, Ser-Gly-Ser-Gly (SGSG) was inserted at the *N*-terminus of the peptide parts of **5-6** to improve their solubility. The results of 27 MHz quartz-crystal microbalance (QCM) measurements of DR5 with **5** and **6** and costaining experiments of Jurkat cells with **5** and anti-DR5 antibody that **5** and **6** bind to DR5 at the different sites from the recognition sites of anti-DR5 antibody. Some cancer cell lines such as Jurkat cells, K562 cells, and Molt-4 cells were stained with **5** for luminescence microscopic observation, showing that Ir complexes exhibit green emitting spots localized inside the cell. In addition, it was turned out that **5** induces cell death of Jurkat cells more slowly than those by **2c-d** (**5** requires almost 24 h to induce considerable cell death, while **2c**, **2d**, **3a,** and **3c** induce cell death in a couple of hours). Binding of **5** to cancer cells and its cytotoxicity against cancer cells are dependent on the DR5 expression level of cancer cells. Further mechanistic studies suggest that cell death induced by **5** is necrotic type. Interestingly, the treatment of cancer cells with **5** and then with anti-DR5 antibody lowers the staining level on cell membrane of Jurkat cells by anti-DR5 antibody, indicating that DR5 undergoes endocytosis upon binding with **5**. Furthermore, it was found that DR5 moved from cytoplasm to the cell membrane or reproduced on the cell membrane after additional incubation for 6 h. Finally, detection of Jurkat cells spiked in bovine blood is demonstrated. To the best of our knowledge, **5** is the first example of artificial luminescent death ligand that detects cancer cells and induces their necrosis-type cell death.

## 2. Experimental Section

### 2.1. General Information

All reagents and solvents were purchased from commercial suppliers and were used without further purification, unless otherwise noted. MTT (3-(4,5-dimethyl-2-thiazolyl)-2,5-diphenyl-2*H*-tetrazolium bromide) was purchased from Dojindo. Z-VAD-FMK (Z-Val-Ala-Asp(OMe) fluoromethylketone) was purchased from the Peptide Institute. Necrostatin-1 and IM-54 were purchased from Enzo Life Sciences. Anti-DR5 antibody [DR5-01-1] (phycoerythrin) (ab55863) was purchased from Abcam. TRAIL/Apo2L (human recombinant), chloroquine diphosphate, verapamil hydrochloride, and nicardipine hydrochloride were purchased from Wako Pure Chemical Industries. The oligomycin complex was purchased from Cayman Chemical Co., quinidine and 4-aminopyridine were purchased from TCI. CCCP (carbonyl cyanide 3-chlorophenylhydrazone), and bafilomycin A1 and amiloride hydrochloride were purchased from Sigma-Aldrich. Propidium iodide and NaN_3_ were purchased from Nacalai Tesque. Annexin V-Cy3 was purchased from BioVision, Inc. All aqueous solutions were prepared using deionized and distilled water. UV spectra were recorded on a JASCO V-550 spectrophotometer, equipped with a temperature controller unit at 25 ± 0.1°C. Emission spectra were recorded on a JASCO FP-6200 and FP-6500 spectrometers. IR spectra were recorded on a Perkin-Elmer FT-IR spectrophotometer (Spectrum100). ^1^H NMR (300 MHz) spectra were recorded on a JEOL Always 300 spectrometer. Tetramethylsilane (TMS) was used as an internal reference for ^1^H measurements in CDCl_3_ and CD_3_OD, and 3-(Trimethylsilyl)-propionic-2,2,3,3-*d*_4_ acid (TSP) sodium salt was used as an external reference for ^1^H NMR measurement in D_2_O. Mass spectral measurements were performed on a JEOL JMS-SX102A and Varian TQ-FT. Luminescence imaging studies were performed using fluorescent microscope (Biorevo, BZ-9000, Keyence). Thin-layer chromatographies (TLC) and silica gel column chromatographies were performed using Merck Art. 5554 (silica gel) TLC plate and Fuji Silysia Chemical FL-100D, respectively. HPLC experiments were carried out using a system consisting of two PU-980 intelligent HPLC pumps (JASCO, Japan), a UV-970 intelligent UV-visible detector (JASCO), a Rheodine injector (Model no. 7125), and a Chromatopak C-R6A (Shimadzu, Japan). For analytical HPLC, the Senshu Pak Pegasil ODS column (Senshu Scientific Co., Ltd.) (4.6*φ *×* *250 mm, No. 07051001) was used. For preparative HPLC, the Senshu Pak Pegasil ODS SP100 column (Senshu Scientific Co., Ltd.) (20*φ *×* *250 mm, No. 1302014G) was used. Lyophilization was performed with the freeze-dryer FD-5N (EYELA).

### 2.2. Synthesis of Ir Complexes and Peptide Units

Ir Complexes **1** and **7**: These complexes were synthesized according to our previously reported procedure [75].

Ir Complex **8**: A solution of Ir complex **7** (50 mg, 0.06 mmol), DIEA (190 *µ*L, 1.08 mmol), and PyBOP (283 mg, 0.54 mmol) in distilled DMF (3 mL) was stirred for 10 min at room temperature, to which *β*-alanine ethyl ester (84 mg, 0.54 mmol) was added. The whole solution was stirred for 18 h and concentrated under reduced pressure. The remaining residue was extracted with CHCl_3_/H_2_O, dried over Na_2_SO_4_, concentrated under reduced pressure, and purified by silica gel column chromatography. (hexanes: AcOEt = 2 : 3 → 1 : 2 → 1 : 4 → 1 : 6) to obtain Ir complex **8** as a yellow solid. This Ir complex (30 mg, 0.026 mmol) and 5 M LiOH (25 mg, 1.06 mmol) in H_2_O/THF (1/1, 9 mL) was stirred at 60°C for 17 h, and then 2 N HCl (pH = 1) was added to form precipitate. The precipitate was filtrated and washed with H_2_O to give Ir complex **8** as a yellow solid (26 mg, 41% from **7**). IR (ATR): *ν* = 3279, 2924, 2579, 1975, 1712, 1586, 1527, 1471, 1259, 1186, 1068, 1022, 892, 781, and 750 cm^−1^. ^1^H NMR (CD_3_OD, 300 MHz): *δ* = 8.04 (d, 3H, *J* = 8.1 Hz), 7.74 (m, 6H), 7.47 (d, 3H, *J* = 5.4 Hz), 6.98 (t, 3H, *J* = 7.5 Hz), 6.69 (s, 3H), 3.59 (t, 6H, *J* = 5.1 Hz), 2.63 (t, 6H, *J* = 6.9 Hz), and 2.12 (s, 9H) ppm. ESI-MS (*m*/*z*): calcd. for C_48_H_45_IrN_6_O_9_ [M]^+^: 1042.28773 and found: 1042.28784.

Ir Complex **9** [72]: DIEA (63 mg, 0.5 mmol), PyBOP (126.6 mg, 0.24 mmol), and mono-Boc-protected hexamethylenediamine (103 mg, 0.5 mmol) were added to a solution of **7** (33 mg, 0.039 mmol) in distilled DMF (1 mL). The reaction mixture was stirred at room temperature for 36 h and then concentrated under reduced pressure. The remaining residue was purified by silica gel column chromatography (CHCl_3_/MeOH, 1/0 to 50/1 to 0/1), gel permeation chromatography (CHCl_3_), and recrystallization from hexanes/CHCl_3_ to afford the Boc-protected **9** as a yellow solid. A mixture of TMSCl (21 mg, 0.46 mmol) and NaI (70 mg, 0.46 mmol) in CH_3_CN (1 mL) was added to a suspension of Boc-protected **9** (21.6 mg, 15 *μ*mol) in CH_3_CN (3.3 mL). The mixture was stirred at room temperature for 10 min and sonicated for 2 min. The insoluble compound was centrifuged and washed with CH_3_CN to give **9** as the HI salt. The product was purified by preparative HPLC (H_2_O (0.1% TFA)/CH_3_CN (0.1% TFA) = 80/20 to 50/50 (30 min), *t*_r_ = 21 min, 6.0 mL/min), followed by lyophilization to give **9** as a yellow solid (41 mg, 62% as 3 TFA salt). IR (ATR): *ν* = 2934, 2862, 2035, 1674, 1600, 1531, 1472, 1425, 1262, 1199, 1069, 892, 781, and 721 cm^−1^. ^1^H NMR (D_2_O, 300 MHz): *δ* = 8.04 (d, 3H, *J* = 8.41 Hz), 7.82–7.76 (m, 6H), 7.69 (d, 3H *J* = 5.4 Hz), 7.09–7.05 (t, 3H *J* = 6.0 Hz), 6.58 (s, 3H), 3.38–3.33 (m, 6H), 3.01–2.96 (t, 6H, *J* = 6.9 Hz), 2.11 (s, 9H), 1.68–1.62 (m, 12H), and 1.43 (m, 12H) ppm. ESI-MS (*m*/*z*): calcd for C_57_H_73_IrN_9_O_3_ [M + H]^+^: 1124.54656 and found: 1124.54487.

Fmoc-Leu-NH-SAL-Trt(2-Cl)-resin: NH_2_-SAL-Trt (2-Cl)-resin (500 mg, 0.54 mmol/g) was suspended in DMF (2.5 mL), to which a mixture of Fmoc-Leu-OH (3 eq.), DIC (4 eq.), and HOBt (4 eq.) was added. After stirring for 2 h, the residue was filtrated, washed with DMF, CH_2_Cl_2_, and ether, and dried in vacuo. The amount of Fmoc-Leu-OH loaded on the resin was determined by UV absorption of the Fmoc derivative at 301 nm (*ε*_301nm_ = 7800 M^−1^·cm^−1^) after treatment with 20% (v/v) piperidine in DMF. Yield: 593 mg (loading: 0.43 mmol/g resin).

Cyclic peptide **CP1**: Fmoc-protecting group of Fmoc-Leu-NH-SAL-Trt(2-Cl)-resin (500 mg, 0.43 mmol) was deprotected by treatment with 20% piperidine in DMF. Each Fmoc-Xaa-OH (4 eq.) was coupled at 45°C for 1 h to the Fmoc-deprotected resin in the presence of DIC (4 eq.) and HOBt (8 eq.) in DMF (2.5 mL). After the last deprotection, the *N*-terminus was acetylated using Ac_2_O (4 eq.) and DIEA (4 eq.) in DMF (2.5 mL). The peptide was cleaved from the resin and also deprotected using a mixture of TFA/H_2_O/TIPS/thioanisole (85/2.5/10/2.5). After 4 h of stirring, the resin was filtered and washed with TFA. After evaporation of TFA, the precipitation was obtained by adding cooled Et_2_O and collected by centrifugation. The crude product was purified by preparative HPLC (H_2_O (0.1% TFA)/CH_3_CN (0.1% TFA) = 80/20→50/50 (30 min) *t*_r_ = 11 min, 1 mL/min), lyophilized to give **CP1** as a linear form. The linear form of **CP1** was subjected to cyclization using 0.1 mM aq. NH_4_HCO_3_ (1 mg/1 mL). After cyclization reaction for 12–24 h, the solution was concentrated and the remaining residue was purified again by preparative HPLC (H_2_O (0.1% TFA)/CH_3_CN (0.1% TFA) = 80/20→50/50 (30 min), *t*_r_ = 7.5 min, 1 mL/min), lyophilized to give **CP1** as white powder (217 mg, 26%). IR (ATR): *ν* = 3217, 2992, 2291, 2167, 2097, 2031, 1980, 1637, 1508, 1434, 1173, 1118, 839, 800, and 723 cm^−1^. ^1^H NMR (D_2_O, 300 MHz): *δ* = 7.62 (m, 1H), 7.46 (m, 1H), 7.23 (m, 2H), 7.22 (m, 1H), 5.59 (s, 1H), 4.29 (m, 17H), 3.91 (s, 1H), 3.34 (s, 1H), 2.80 (s, 8H), 2.55 (m, 3H), 2.49 (m, 3H), 2.34 (m, 6H), 1.95 (m, 5H), 1.59 (m, 7H), 1.41 (m, 31H), 1.29 (m, 2H), and 0.98 (m, 50H) ppm. ESI-MS (*m/z*): calcd for C_85_H_142_N_30_O_23_S_6_ [M + 2H]^2+^: 1008.01809 Found: 1008.01709.

Cyclic peptides **CP2** and **CP3** were prepared according to the same procedure described for cyclic peptide **CP1**.

Cyclic peptide **CP2**: white powder (371 mg, 29% over two steps). HPLC: (H_2_O (0.1% TFA)/CH_3_CN (0.1% TFA) = 90/10→60/40 (30 min), *t*_r_ = 16 min, 1 mL/min). IR (ATR): *ν* = 3283, 2939, 2167, 2027, 1980, 1651, 1533, 1431, 1177, 1119, 1044, 891, 840, 798, 721, and 655 cm^−1^. ^1^H NMR (D_2_O, 300 MHz): *δ* = 7.49 (m, 1H), 7.38 (m, 1H), 7.20 (m, 2H), 7.09 (m, 1H), 4.97 (m, 24H), 3.86 (m, 12H), 3.17 (m, 24H), 2.09 (m, 24H), 1.62 (m, 29H), and 0.90 (m, 38H) ppm. ESI-MS (*m*/*z*): calcd. for C_95_H_159_N_34_O_29_S_2_ [M + 3H]^3+^: 768.71559 and found: 768.71568.

Cyclic peptide **CP3**: white powder (18 mg, 23% over two steps). HPLC: (H_2_O (0.1% TFA)/CH_3_CN (0.1% TFA) = 90/10→60/40 (30 min), *t*_r_ = 11 min, 1 mL/min). IR (ATR): *ν* = 3287, 3071, 2965, 2547, 2045, 1778, 1651, 1532, 1440, 1290, 1155, 1042, 845, 700, and 578 cm^−1^. ^1^H NMR (D_2_O, 300 MHz): *δ* = 7.51 (m, 1H), 7.38 (m, 1H), 7.20 (m, 2H), 7.10 (m, 1H), 4.97 (m, 31H), 3.86 (m, 18H), 3.17 (m, 27H), 2.09 (m, 24H), 1.62 (m, 29H), and 0.90 (m, 38H) ppm. ESI-MS (*m/z*): calcd for C_105_H_175_N_38_O_35_S_2_ [M + 3H]^3+^: 864.41581 and found: 864.41735.

Ir Complex **4**: EDC (832 mg, 4.34 mmol) and NHS (500 mg, 4.34 mmol) was added to a solution of **7** (120 mg, 0.14 mmol) in DMF (12 mL). The reaction mixture was stirred for 24 h at room temperature and concentrated under reduced pressure. After addition of CHCl_3_, organic layer was washed with sat. NH_4_Cl. The combined organic layer was washed with H_2_O, dried over Na_2_SO_4_, filtered, and concentrated under reduced pressure to give NHS ester of **7** as a yellow solid (110 mg, 67%). IR (ATR): *ν* = 2941, 2162, 1705, 1581, 1519, 1380, 1293, 1206, 1066, 976, 886, and 648 cm^−1^. ^1^H-NMR (CDCl_3_, 300 MHz): *δ* = 8.45 (s, 3H), 8.00 (d, 3H, *J* = 7.8), 7.73 (t, 3H, *J* = 7.8), 7.42 (d, 3H, *J* = 5.7), 6.97 (t, 3H, *J* = 6.0), 6.82 (s, 3H), 2.89 (s, 12H), and 2.42 (s, 9H) ppm. ESI-MS (*m/z*): calcd for C_51_H_39_IrN_6_O_12_ [M]^+^: 1120.22552. Found: 1120.22641. NHS ester of Ir complex **7** (1 mg, 0.8 *µ*mol) was added to a solution of **CP1** (5.39 mg, 2.6 *µ*mol) and DIEA (4.7 *µ*L, 0.026 mmol) in DMF (100 *μ*L) and stirred for 24 h at room temperature in the dark. After that, 0.1% TFA H_2_O was added to the reaction mixture and the crude product was purified by preparative HPLC (H_2_O (0.1% TFA)/CH_3_CN (0.1% TFA) = 80/20→50/50 (30 min), *t*_r_ = 21 min, (1 mL/min)), lyophilized to give **4** as a yellow powder (3.8 mg, 43% from **7**). IR (ATR): *ν* = 32879, 2320, 1981, 1638, 1535, 1426, 1264, 1201, 1134, 923, 835, 800, and 722 cm^−1^. ^1^H NMR (DMSO, 300 MHz): *δ* = 8.26 (s, 3H), 8.22 (s, 7H), 8.15 (m, 10H), 7.83 (m, 8H), 7.40 (m, 8H), 7.30 (m, 12H), 7.13 (m, 21H), 6.96 (s, 13H), 6.55 (s, 3H), 4.23 (m, 6H), 4.12 (m, 14H), 3.11 (s, 3H), 2.51 (m, 29H), 2.49 (m, 10H), 2.48 (m, 24H), 2.22 (m, 12H), 1.76 (s, 7H), 1.47 (m, 36H), 1.3 (s, 3H), and 0.80 (m, 41) ppm. ESI-MS (*m/z*); calcd for C_294_H_450_IrN_93_O_72_S_6_ [M + 6H]^6+^: 1137.2104 and found: 1137.20847.

Ir Complex **5**: EDC (137 mg, 0.71 mmol) and NHS (83 mg, 0.71 mmol) was added to a solution of **8** (25 mg, 0.023 mmol) in DMF (2.5 mL). The resulting solution was stirred for 24 h at room temperature. The reaction mixture was concentrated under reduced pressure. After addition of CHCl_3_, the organic layer was washed with sat. NH_4_Cl. The combined organic layer was washed with H_2_O, and then dried over Na_2_SO_4_, filtered, and concentrated under reduced pressure to give NHS ester of **8** as a yellow solid (20 mg, 62%). IR (ATR): *ν* = 3743, 3318, 2925, 1815, 1780, 1706, 1471, 1375, 1260, 1204, 1131, 1067, 994, 781, and 648 cm^−1^. ^1^H-NMR (CDCL_3_, 300 MHz): *δ* 7.94 (d, 3H, *J* = 8.1), 7.73 (s, 3H), 7.58 (t, 3H, *J* = 7.8), 7.40 (d, 3H, *J* = 5.1), 6.84 (t, 3H, *J* = 6.3), 6.67 (s, 3H), 6.50 (t, 3H, *J* = 6.6), 3.81 (d, 6H, *J* = 5.1), 2.95 (t, 6H, *J* = 6.3), 2.91 (s, 12H), and 2.23 (s, 9H). ESI-MS (*m/z*): calcd for C_60_H_54_IrN_9_O_15_ [M]^+^: 1333.33686 and found: 1333.33747. NHS ester of Ir complex **8** (6 mg, 0.0044 mmol) was added to a solution of **CP2** (31.06 mg, 0.013 mmol) and DIEA (23 *µ*L, 0.134 mmol) in DMF (600 *μ*L) and stirred for 24 h at room temperature in the dark. The reaction mixture was diluted with 0.1% TFA H_2_O and purified by preparative HPLC (H_2_O (0.1% TFA)/CH_3_CN (0.1% TFA) = 80/20→50/50 (30 min), *t*_r_ = 10 min, 1 mL/min), lyophilized to give **5** as a yellow powder (15.45 mg, 27% from **8**). IR (ATR): *ν* = 3282, 3074, 2964, 2054, 1980, 1639, 1531, 1472, 1425, 1261, 1181, 915, 799, and 720 cm^−1^. ^1^H NMR. (D_2_O, 300 MHz): *δ* = 7.68 (s, 3H), 7.46 (s, 3H), 7.08 (m, 6H), 6.89 (m, 3H), 6.68 (s, 3H), 3.79 (m, 18H), 3.73 (m, 7H), 3.71 (m, 11H), 3.25 (m, 18H), 3.23 (m, 12H), 3.18 (m, 13H), 2.73 (m, 5H), 2.24 (m, 193H), 2.23 (m, 20H), 2.00 (m, 11H), 1.63 (m, 45), 1.35 (m, 50H) 1.15 (m, 12H), and 0.89 (m, 74H) ppm. ESI-MS (*m/z*): calcd. for C_333_H_513_IrN_108_O_93_S_6_ [M + 6H]^6+^: 1316.94104. Found: 1316.94569.

Ir complex **6** was prepared according to the same procedure described for **5**.

Ir Complex **6**: yellow powder (8.3 mg, 21% from **8**). HPLC: (H_2_O (0.1% TFA)/CH_3_CN (0.1% TFA) = 90/10→60/40 (30 min), *t*_r _= 12 min, 1 mL/min). IR (ATR): *ν* = 3383, 2963, 2014, 1984, 1638, 1535, 1475, 1262, 1200, 1057, 836, 799, and 720 cm^−1^. ^1^H NMR (D_2_O, 300 MHz): *δ* = 7.72 (s, 3H), 7.42 (s, 3H), 7.17 (m, 6H), 6.95 (m, 3H), 6.78 (s, 3H), 3.86 (m, 23H), 3.71 (m, 38H), 3.23 (m, 42H), 2.73 (m, 31H), 2.07 (m, 12H), 1.92 (m, 70H), 1.62 (m, 69H), 1.34 (m, 132H), and 0.88 (m, 120H) ppm. ESI-MS (*m/z*): calcd for C_363_H_563_IrN_120_O_111_S_6_ [M + 8H]^8+^: 1096.00145 and found: 1096.00136.

### 2.3. UV/Vis Absorption and Luminescence Spectra Measurements

UV/Vis spectra were recorded on a JASCO V-550 UV/Vis spectrophotometer equipped with a temperature controller, and emission spectra were recorded on a JASCO FP-6200 spectrofluorometer at 25°C. Before the luminescence measurements, sample aqueous solutions were degassed by Ar bubbling for 10 min in quartz cuvettes equipped with Teflon septum screw caps. Concentrations of all the Ir complexes in stock solutions (DMSO) were determined based on a molar extinction coefficient of 380 nm (*ε*_380nm_ = 1.08 ±0.07 × 10^4^ M^−1^·cm^−1^). Quantum yields for luminescence (Φ) were determined by comparing with the integrated corrected emission spectrum of a quinine sulfate standard, whose emission quantum yield in 0.1 M H_2_SO_4_ was assumed to be 0.55 (excitation at 366 nm). Equation (1) was used to calculate the emission quantum yields, in which Φ_s_ and Φ_r_ denote the quantum yields of the sample and reference compounds, *η*_s_ and *η*_r_ are the refractive indexes of the solvents used for the measurements of the sample and reference, *A*_s_ and *A*_r_ are the absorbance of the sample and the reference, and *I*_s_ and *I*_r_ stand for the integrated areas under the emission spectra of the sample and reference, respectively (all of the Ir compounds were excited at 366 nm for luminescence measurements in this study):

(1) $$\begin{array}{c} \Phi_{s}=\frac{\Phi_{\text{r}}\left(\eta_{s}^{2}A_{\text{r}}I_{\text{s}}\right)}{\left(\eta_{r}^{2}A_{\text{s}}I_{\text{r}}\right)}. \end{array}$$

The luminescence lifetimes of sample solutions were measured on a TSP1000-M-PL (Unisoku, Osaka, Japan) instrument by using THG (355 nm) of Nd:YAG laser, Minilite I (Continuum, CA, USA), at 25°C in degassed aqueous solutions. The R2949 photomultiplier were used to monitor the signals. Data were analyzed using the nonlinear least-squares procedure.

### 2.4. 27 MHz Quartz Crystal Microbalance (QCM) Analysis

QCM analysis was performed on an Affinix-Q4 apparatus (Initium Inc., Japan). The clean Au (4.9 mm^2^) electrode equipped on the quartz crystal was incubated with an aqueous solution of 3,3′-dithiodipropionic acid (3 mM, 4 *μ*L) at room temperature for 60 min. After washing with distilled water, the surface was activated by a mixture of EDC·HCl (0.52 M) and *N*-hydroxy succinimide (0.87 M) for 30 min, washed with distilled water, and then treated with DR5 (100 *μ*g/mL, 4 *μ*L) at room temperature for 60 min. After washing with distilled water, an aqueous solution of 1 M ethanolamine (5 *μ*L) was added as a blocking reagent. After washing with distilled water, cell was filled with phosphate-buffered saline (PBS) (500 *μ*L). The apparent binding constants (*K*_app_) for IPHs with DR5 in PBS were calculated from the decrease in frequency. The nonspecific response was subtracted from frequency decrease curve to obtain apparent complexation constants *K*_app_ and dissociation constants *K*_d_ (=1/*K*_app_).

### 2.5. Cell Culture

All cell lines (Jurkat, Molt-4, and K562 cells) were cultured in RPMI 1640 medium supplemented with 10% heat-inactivated fetal calf serum (FCS), L-glutamine, HEPES (2-[4-(2-hydroxyethyl)-1-piperazinyl]ethanesulfonic acid, p*K*_a_ = 7.5), 2-mercaptoethanol, and penicillin/streptomycin and monothioglycerol (MTG) in a humidified 5% CO_2_ incubator at 37°C.

### 2.6. MTT Assay

Jurkat cells (1.0 × 10^5^ cells/mL) were incubated in 1% DMSO, 10% FCS RPMI 1640 medium (MTG free) containing solution of Ir complexes **4–6** (0–75 *µ*M) under 5% CO_2_ at 37°C for 1 to 24 h in 96-well plates (BD Falcon), then 0.5% MTT reagent in PBS buffer (10 *µ*L) was added to the cells. After incubation under 5% CO_2_ at 37°C for 4 h, formazan lysis solution (10% SDS in 0.01 N HCl) (100 *µ*L) was added and incubated overnight under same conditions, followed by measurement of absorbance at 570 nm by a microplate reader (Bio-Rad). MTT assay of Molt-4 cells and K562 cells with Ir complexes was also performed according to the same procedure described above.

### 2.7. MTT Assay in the Presence of Caspase Inhibitor (Z-VAD-FMK) and Necroptosis Inhibitor (Necrostatin-1) and Oxidative Stress Induce Necrosis Inhibitor (IM-54)

Jurkat cells (1.0 × 10^5^ cells/mL) were incubated in 10% FCS RPMI 1640 medium (MTG-free) containing a solution Z-VAD-fmk (15 *µ*M)/necrostatin-1 (30 *µ*M)/IM-54 (10 *µ*M)/(under 5% CO_2_ at 37°C for 1 h in 96-well plates (BD Falcon). Then, Ir complexes (75 *µ*M) were added and incubated under 5% CO_2_ at 37°C for 24 h, and then 0.5% MTT reagent in PBS buffer (10 *µ*L) was added to the cells and incubated under 5% CO_2_ at 37°C for 4 h. A formazan lysis solution (10% SDS in 0.01 N HCl) (100 *µ*L) was added, and the resulting solution incubated overnight under the same conditions, followed by measurement of absorbance at 570 nm with a microplate reader (Bio-Rad).

### 2.8. Fluorescent Microscopy Studies of Jurkat Cells, K562 Cells, and Molt-4 Cells with Ir Complexes

Jurkat cells, K562 cells, or Molt-4 cells (1.0 × 10^6^ cells/mL) were incubated in the absence or presence of Ir complexes in 10% FCS RPMI 1640 medium (MTG free) for specified time under 5% CO_2_ at 37°C. The cells were then washed twice with ice-cold PBS with 0.1% NaN_3_ and 0.5% FCS and taken on a Greiner CELLview™ petri dish (35 × 10 mm) and mounted on fluorescent microscope for observation (Biorevo, BZ-9000, Keyence) (excitation: 377 ± 25 nm; emission: 520 ± 35 nm; FF01 filter).

### 2.9. Fluorescent Microscopy Studies of Jurkat Cells with Ir Complexes in the Presence of Inhibitors

Jurkat cells (1.0 × 10^6^ cells/mL) were incubated with the given inhibitors in RPMI 1640 medium (MTG free) with 10% FCS under 5% CO_2_ at 37°C for 1 h, and then Ir complexes were added and then again incubated at 37°C for 24 h. The cells were then washed twice with ice-cold PBS containing 0.5% FCS and 0.1% NaN_3_ and incubated with PI in PBS at room temperature for 10–15 min. The cells were then again washed with PBS buffer and observed by fluorescent microscope (Biorevo, BZ-9000, Keyence) (excitation: 377 ± 25 nm; emission: 520 ± 35 nm; FF01 filter).

### 2.10. Propidium Iodide (PI) Staining

The cells were incubated with Ir complexes for specified time, washed with PBS buffer, and then incubated with PI in PBS buffer at room temperature for 10–15 minutes. The cells were then again washed with PBS buffer and observed by fluorescent microscope (Biorevo, BZ-9000, Keyence) (excitation: 540 ±25 nm; emission: 605 ± 55 nm; TRICT filter) or analyzed by flow cytometer (Beckman Coulter Gallios Flow Cytometer, detector: FL2, excitation: 488 nm, emission: 575 ± 20 nm).

### 2.11. Anti-DR5 Antibody Staining

Given cells were incubated with anti-DR5 antibody at 4°C for 15 min on ice. The cells were then washed twice with ice-cold PBS containing 0.5% FCS and 0.1 % NaN_3_ and were mounted on fluorescent microscope (Biorevo, BZ-9000, Keyence) (excitation: 540 ± 25 nm; emission: 605 ± 55 nm; TRICT filter) or analyzed by flow cytometer (Beckman Coulter Gallios Flow Cytometer, detector: FL2, excitation: 488 nm; emission: 575 ± 20 nm).

### 2.12. Annexin V-Cy3 Staining

Jurkat cells were incubated with Ir complexes for 2 h, washed with PBS buffer, and then suspended in 1X binding buffer. Annexin V-Cy3 was added to the cell suspension and then incubated at room temperature in the dark for 5–10 minutes. The cells were then washed with PBS buffer and observed by fluorescent microscope (Biorevo, BZ-9000, Keyence) (excitation 540 ± 25 nm, emission 605 ± 55 nm, TRICT filter).

### 2.13. Flow Cytometry Analysis of Staining and Cell Death Induction Assay

Jurkat cells, K562 cells, or Molt-4 cells (3.0 × 10^5^ cells) were incubated in the absence or the presence of Ir complexes in 10% FCS RPMI 1640 medium (MTG free) for the specified time under 5% CO_2_ at 37°C. After that, the cells were washed twice with ice-cold FACS buffer and then suspended in 450 *µ*l FACS buffer. The cells were analyzed by flow cytometer (Beckman Coulter Gallios Flow Cytometer, detector: FL2, excitation: 488 nm, emission: 575 ± 20 nm) to detect PI staining or anti-DR5 antibody staining (detector: FL10, excitation: 405 nm, emission: 550 ± 40 nm) to detect Ir complexes staining).

## 3. Results and Discussion

### 3.1. Design and Synthesis of Ir Complex-Peptide Hybrids (IPHs)

**Figure 3:**
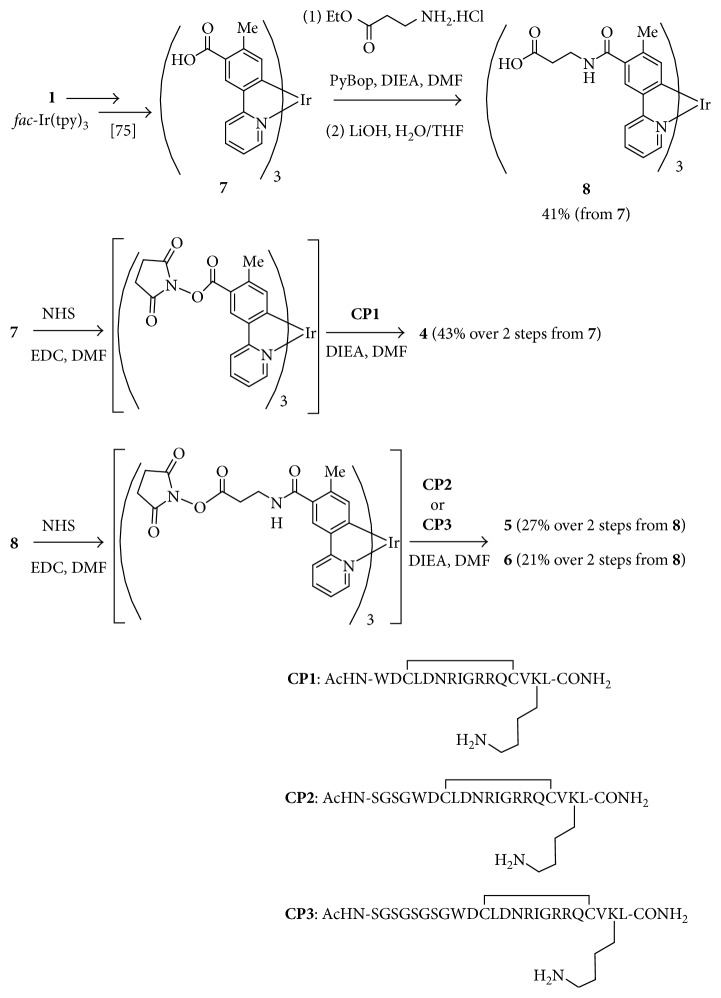
Synthesis of the Ir complex-peptide hybrids (IPHs).

Synthesis of the Ir complex-peptide hybrids (IPHs) **4**–**6** is shown in Figure 3. The Vilsmeier reaction of **1** (*fac*-Ir(tpy)_3_) and the following Pinnick oxidation [72, 73, 75] gave **7**. Condensation of **7** with *β*-alanine ethyl ester hydrochloride and the following ester hydrolysis yielded **8**. Both **7** and **8** were converted to the corresponding *N*-hydroxy succinimide (NHS) esters and then reacted with the peptide units, **CP1**, **CP2**, and **CP3** that had been prepared by Fmoc solid-phase peptide synthesis, to afford **4**–**6**, respectively. Because **4** is poorly soluble in water, a hydrophilic Ser-Gly-Ser-Gly (H_2_N-SGSG-CO) sequence was incorporated to the *N*-terminus of cyclic peptide **CP1** to afford **CP2-3**. All the Ir complexes **4**–**6** were purified by reversed-phase HPLC column with a continuous gradient of H_2_O (0.1%TFA)/CH_3_CN (0.1%TFA) and lyophilized to give yellow powders as the corresponding TFA salts. It should be mentioned that negligible conversion of the facial form of **4**–**6** to the corresponding meridional form was observed during their synthesis and the following biological assays.

### 3.2. UV/Vis and Luminescence Spectra of IPHs

**Figure 4:**
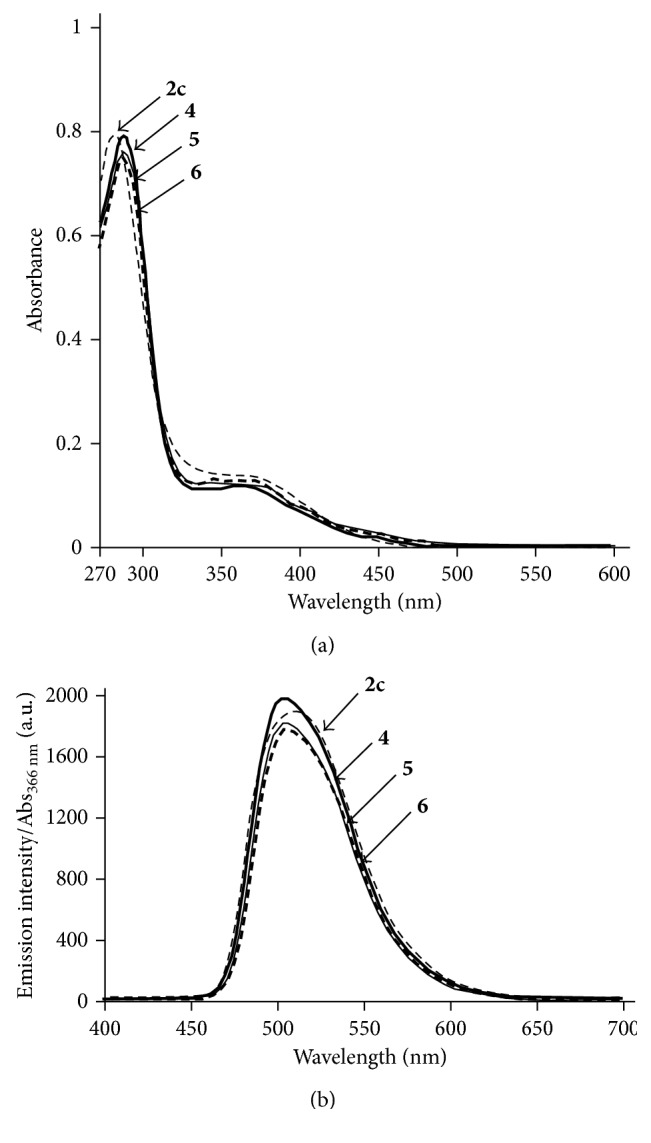
UV/Vis spectra of (a) **2c** (dashed curve), **4** (bold curve), **5** (plain curve), and **6** (bold dashed curve). Emission spectra of (b) **2c** (dashed curve), **4** (bold curve), **5** (plain curve), and **6** (bold dashed curve), in degassed DMSO at 25°C ([Ir complex] = 10 *μ*M, excitation at 366 nm) (a.u. is the arbitrary unit).

UV/Vis and luminescence spectra of Ir complexes **2c**, **4**, **5**, and **6** (10 *µ*M) in DMSO at 25°C are shown in Figure 4, and their photophysical data are summarized in Table 1. The concentrations of the Ir complexes in stock solutions (DMSO) were determined by the molar extinction coefficient at 380 nm (*ɛ*_380nm_ = 1.43 ± 0.03 × 10^4^·M^−1^·cm^−1^) of **4**, **5,** and **6**, which are almost identical to those of typical Ir(tpy)_3_ derivatives having peptides **2a**–**f** and **3a**–**c** (Figure 1), as we previously reported [72, 73]. The strong absorption bands at 270–300 nm were assigned to the ^1^ *π*-*π* ^∗^ transition of tpy ligands and weak shoulder bands at 320–450 nm were assigned as spin-allowed singlet-to-singlet metal-to-ligand charge transfer (^1^MLCT) transitions, spin-forbidden singlet-to-triplet (^3^MLCT) transitions, and ^3^ *π*-*π* ^∗^ transitions. Strong green emission of **4**, **5,** and **6** is observed with emission maxima at ca. 506 nm, which are almost same as that of **2c** (Figure 4(b)). The luminescence quantum yields (Φ) of **4**, **5,** and **6** were determined to be 0.39, 0.33, and 0.36, respectively, and their luminescence lifetimes (*τ*) are 1.1–1.3 *µ*s, which are almost same as those of **1**–**3**.

### 3.3. Determination of Complexation Properties of IPHs with DR5

**Figure 5:**
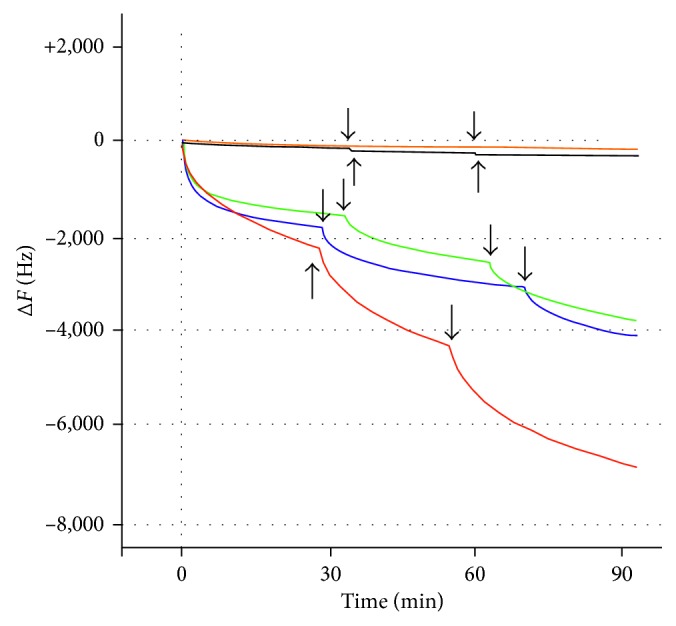
Time course of frequency change (ΔF (Hz)) of IPHs-DR5 complexation. Conditions: temperature, 25°C; solvent, phosphate-buffered saline (PBS). An aliquot of solutions of TRAIL (red curve) (200 *µ*g/mL), **5** (green curve), **6** (blue curve), **9** (orange curve), and **2c** (black curve) ([Ir complex] = 10 mM solution in DMSO) was added to DR5 fixed on the sensor chip. Plain arrows indicate the time when solutions of these analytes were added to DR5.

**Figure 6:**
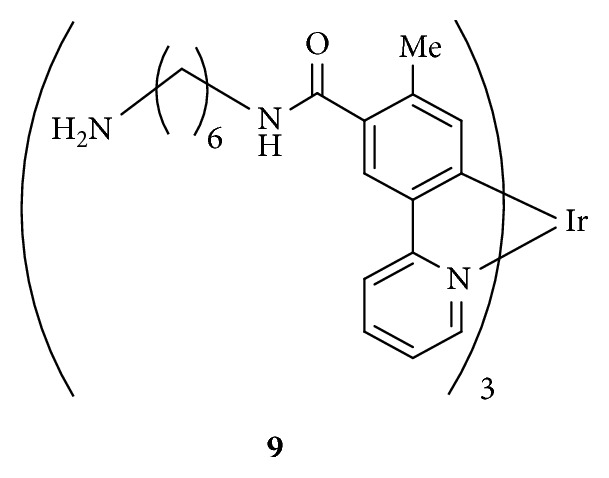
Ir complex having no peptide.

First, complexation of IPHs with DR5 was checked by 27 MHz quartz-crystal microbalance (QCM). DR5 was immobilized on a sensor chip, to which IPHs were added on a certain time interval. Upon addition of IPHs, frequency change was observed (Figure 5) indicating the interaction of IPHs with DR5. The apparent complexation constants (*K*_app_) (and dissociation constant, *K*_d_) for TRAIL, **5**, and **6** with DR5 were determined to be (2.3 ± 0.05) × 10^8^ M^−1^ (*K*_d_ = 4.3 ± 0.1 nM), (3.8 ± 0.1) × 10^5^ M^−1^ (*K*_d_ = 2.7 ± 0.1 *µ*M), and (4.0 ± 0.2) × 10^5^ M^−1^ (*K*_d_ = 2.5 ± 0.1 *µ*M), respectively, assuming 1 : 1 complexation (Table 2). Negligible interaction was observed for **9**, which lacks the receptor-binding peptide (Figure 6) and **2c** that contains a KKGG peptide (Figure 1) [72, 73].

### 3.4. Cancer Cell Death Induced by IPHs, as Evaluated by MTT Assay and Fluorescence Microscopy

**Figure 7:**
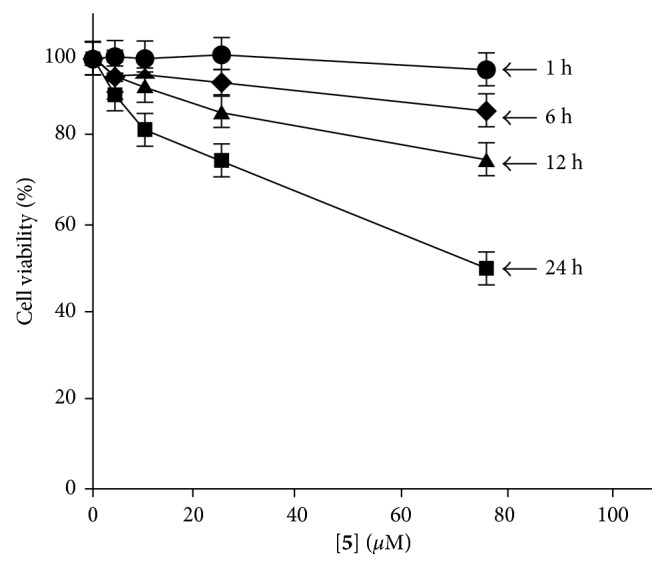
The results of MTT assay: cell viability of Jurkat cells after incubation in the presence of **5** (5–75 *µ*M) for 1 h (filled circles), 6 h (filled diamonds), 12 h (filled triangles), and 24 h (filled squares) at 37°C.

**Figure 8:**
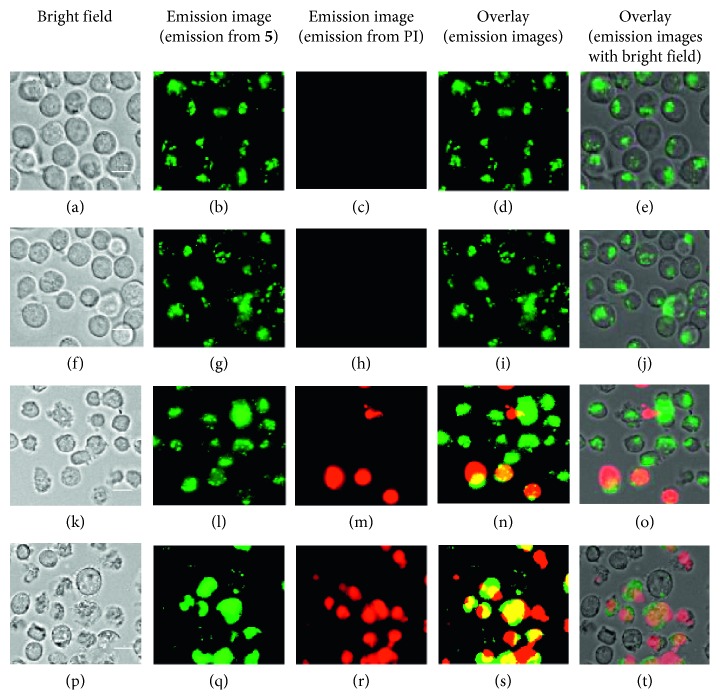
Time lapse luminescence microscopy images (Biorevo, BZ-9000, Keyence) of Jurkat cells (×40) treated with **5** (75 *µ*M) at 37°C. Cell deaths were confirmed by staining with propidium iodide (PI), (a–e) after incubation for 1 h, (f–j) after incubation for 6 h, (k–o) after incubation for 12 h, and (p–t) after incubation for 24 h. Scale bar (white) = 10 *µ*m.

Next, cell death inducing activity of Ir complexes **4**–**6** against Jurkat cells was evaluated by MTT assay (MTT = 3-(4,5-dimethyl-2-thiazolyl)-2,5-diphenyl-2H-tetrazolium bromide). Jurkat cells were incubated with the given concentrations of **5** or **6** in 10% FCS (fetal calf serum) RPMI 1640 (MTG free) medium at 37°C and treated with MTT reagent. It was observed that the induction of cell death of Jurkat cells by **5** and **6** is much slower than that by our previous Ir complexes **2c**, **2d,** and **3c**, which induce cell death of Jurkat cells in 1 h. Because both **5** and **6** interact with DR5 to the same extent, as described in Figure 5 and Table 2, the following assays were carried out with **5**. Jurkat cells were observed by luminescence microscopy after incubation with **5** for 1 h, 6 h, 12 h, and 24 h and stained with propidium iodide (PI) to check their cell death. As summarized in Figures 7 and 8, **5** requires ca. 24 h to induce considerable cell death (ca. 50% cell death at [**5**] = 72 *µ*M). For comparison, negligible **4**-induced cell death, even after incubation for 16 h, was possibly due to its low solubility in water (Figure S1 in Supplementary Materials).

### 3.5. Luminescence Staining of Jurkat Cells with IPHs

**Figure 9:**
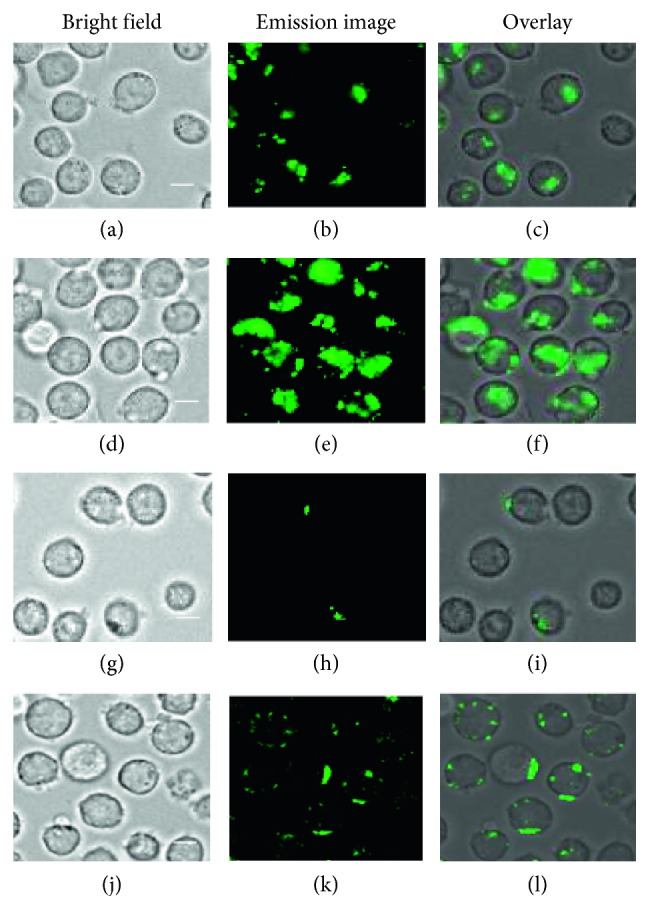
Luminescence microscopy images (Biorevo, BZ-9000, Keyence) of Jurkat cells (×40) stained with **5**. (a–c) Jurkat cells after incubation with **5** (5 *µ*M) at 37°C for 1 h; (d–f) Jurkat cells after incubation with **5** (10 *µ*M) at 37°C for 1 h; (g–i) Jurkat cells after incubation with **5** (20 *µ*M) at 4°C for 1 h; (j–l) Jurkat cells after incubation with NaN_3_ (5 mM) at 4°C for 15 min and then with **5** (5 *µ*M) at 37°C for 1 h. Scale bar (white) = 10 *µ*m.

Slow induction of cell death by IPHs allowed us to conduct luminescence staining experiments of Jurkat cells. Figures 9–9 and 9–9 indicate that **5** (5 and 10 *µ*M) binds to Jurkat cells after incubation at 37°C for 1 h. Passive uptake of **5** through the cell membrane is unlikely, due to the facts that negligible emission was observed after incubation at 4°C for 1 h ([**5**] = 20 *µ*M) (Figures 9–9) and that the pretreatment with NaN_3_ (a metabolic inhibitor) at 4°C for 15 min ([NaN_3_] = 5 mM, a concentration required to inhibit metabolic activity) exhibits the localization of **5** on the cell membrane (Figures 9–9).

Competitive staining of Jurkat cells with **5** and DR5 binding peptide [20–22] (**CP1**, Figure 3) was conducted. Jurkat cells were incubated with **CP1** for 1 h at 37°C to which **5** was added and incubated again for 1 h at 37°C. Green emission from the cells was considerably reduced by the pretreatment with **CP1** (100 *µ*M) than those treated with only **5** (Figure S2 in Supplementary Materials). Similar results were obtained by flow cytometry (Figure S3 in Supplementary Materials), implying that **5** competes with **CP1** for DR5.

### 3.6. Costaining of Jurkat Cells by IPHs and Anti-DR5 Antibody and Observation of the Movement of DR5 between Cell Surface and Cytoplasm

**Figure 10:**
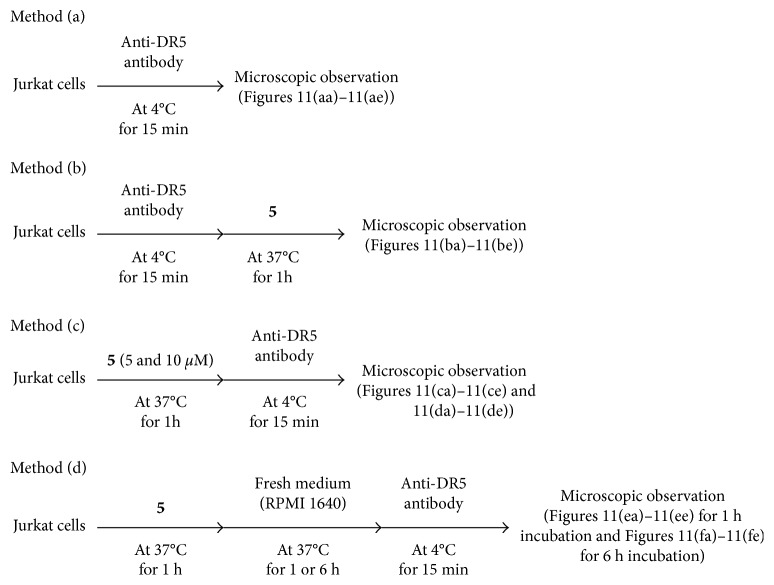
Costaining assay protocol.

**Figure 11:**
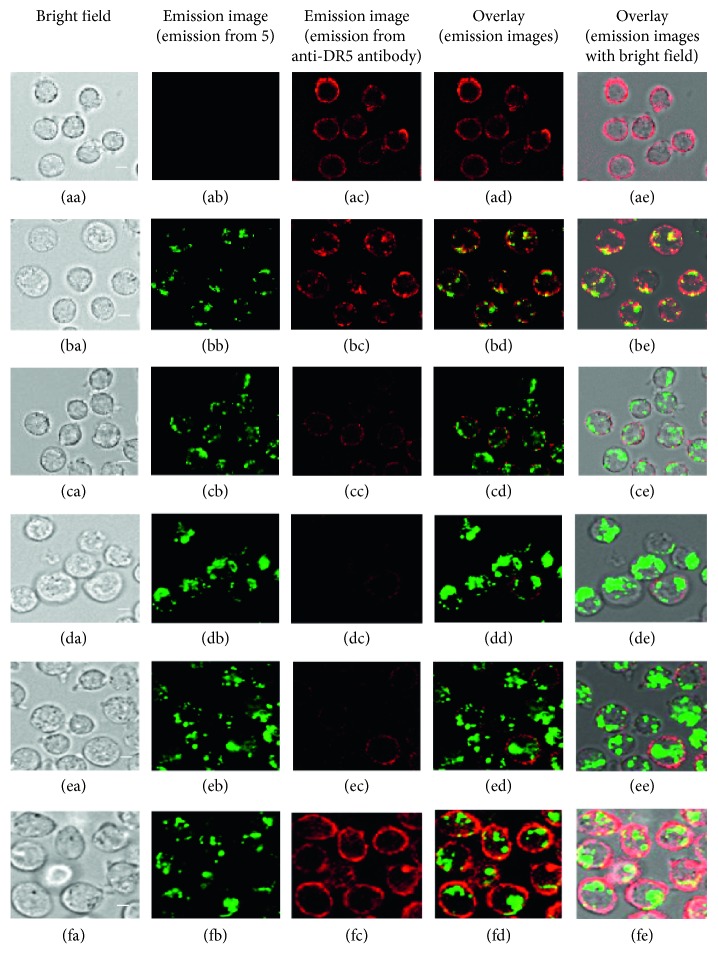
Luminescence microscopic images of (Biorevo, BZ-9000, Keyence) of Jurkat cells stained with **5** and anti-DR5 antibody obtained by protocol presented in Figure 10. (aa–ae) Jurkat cells incubated with anti-DR5 antibody (15 *µ*g/mL) at 4°C for 15 min. (ba–be) Jurkat cells incubated with anti-DR5 antibody (15 *µ*g/mL) at 4°C for 15 min and then with **5** (5 *µ*M) at 37°C for 1 h. (ca–ce) Jurkat cells incubated with **5** (5 *µ*M) at 37°C for 1 h and then with anti-DR5 antibody (15 *µ*g/mL) at 4°C for 15 min. (da–de) Jurkat cells incubated with **5** (10 *µ*M) at 37°C for 1 h and then with anti-DR5 antibody (15 *µ*g/mL) at 4°C for 15 min. (ea–ee) Jurkat cells incubated with **5** (10 *µ*M) at 37°C for 1 h and then in fresh medium for 1 h (2 h in total) and then with anti-DR5 antibody (15 *µ*g/mL) at 4°C for 15 min. (fa–fe) Jurkat cells incubated with **5** (10 *µ*M) at 37°C for 1 h and then in fresh medium for 6 h (7 h in total) and then with anti-DR5 antibody (15 *µ*g/mL) at 4°C for 15 min. Scale bar (white) = 10 *µ*m.

In Figures 11–11, red emission was observed on the cell membrane of Jurkat cells that were treated with anti-DR5 antibody (conjugated with a red color fluorochrome) at 4°C for 15 min (Method a in Figure 10). Next, we observed luminescence images of Jurkat cells that were treated with anti-DR5 antibody at 4°C for 15 min and then incubated with **5** at 37°C for 1 h (Method b in Figure 10). In Figures 11 and 11, green emission from **5** and red emission from anti-DR5 antibody were observed, respectively. The overlays of Figures 11 and 11 show yellow spots (Figures 11 and 11), suggesting that anti-DR5 antibody and **5** stain the same or similar area on the cells. Besides, it was found that there is negligible difference between emission intensity (from anti-DR5 antibody) of Jurkat cells treated with only anti-DR5 antibody and then **5** (Figure S4 in Supplementary Materials), suggesting that both of **5** and anti-DR5 antibody bind with DR5, possibly at the different sites.

Next, the order of the treatment of Jurkat cells with anti-DR5 antibody and **5** was reversed. In this experiment, Jurkat cells were treated with **5** (5 *µ*M) and then with anti-DR5 antibody (Method c in Figure 10). In this case, very weak red emission was observed from Jurkat cells (Figures 11–11), suggesting that the expression level of DR5 on cell membrane was decreased by the treatment with **5**. The increase in the concentration of **5** to 10 *µ*M resulted in the considerable decrease in the red emission from anti-DR5 antibody (Figures 11–11). Similar results were observed in flow cytometry assay, in which Jurkat cells treated with only anti-DR5 antibody exhibit high emission intensity (red curve in Figure S5 in Supplementary Materials).

In the previous experiments, it was hypothesized that DR5 is internalized from the cell membrane to the cell cytoplasm after binding with IPH. To check this hypothesis, Jurkat cells were incubated with **5** at 37°C for 1 h, washed with fresh medium, and incubated again in fresh medium at 37°C for 1 or 6 h (Method d in Figure 10) (Figures 11–11 for 1 h incubation and Figures 11–11 for 6 h incubation). Interestingly, the red emission from anti-DR5 antibody was restored after an additional incubation for 6 h, as displayed in Figures 11–11, showing a good contrast to Figures 11–11 obtained without incubation in fresh medium for 6 h. In flow cytometry assay, red emission intensity from anti-DR5 antibody in cells was reduced after 1 h incubation with **5** and then anti-DR5 antibody in comparison to Jurkat cells treated with only anti-DR5 antibody. After incubation of Jurkat cells with **5** and then again in fresh medium for 6 h, emission from anti-DR5 antibody was restored (Figure S6 in Supplementary Materials).

**Figure 12:**
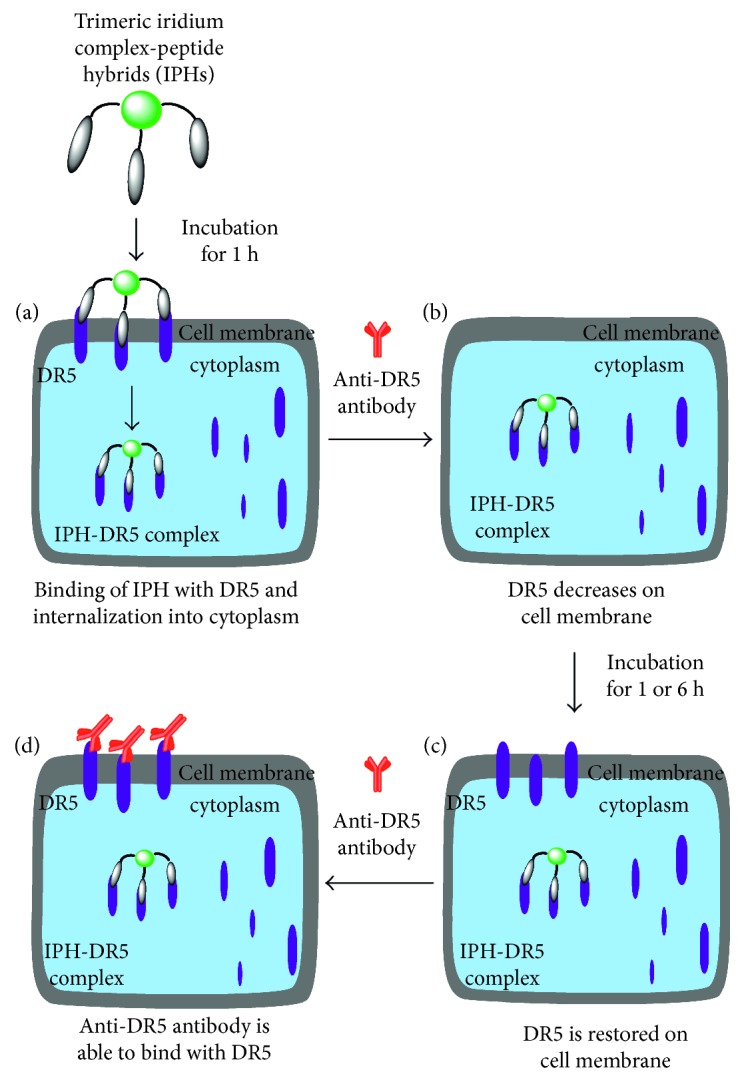
Schematic presentation of the behavior of DR5 after complexation with IPH.

Our assumption based on the results of these experiments is summarized in Figure 12. (i) Upon incubation of Jurkat cells with IPH at 37°C for 1 h, IPH-DR5 complex undergoes internalization into the cytoplasm from the cell surface (Figure 12). (ii) As a result, the binding of anti-DR5 antibody to the cell membrane becomes weak (Figure 12). (iii) After incubation of Jurkat cells (in which IPH-DR5 complexes is internalized in the cytoplasm) in fresh medium at 37°C for 1 or 6 h, a considerable amount of DR5 is restored on the cell membrane (Figure 12), and then (iv) anti-DR5 antibody is able to bind DR5 (Figure 12).

**Figure 13:**
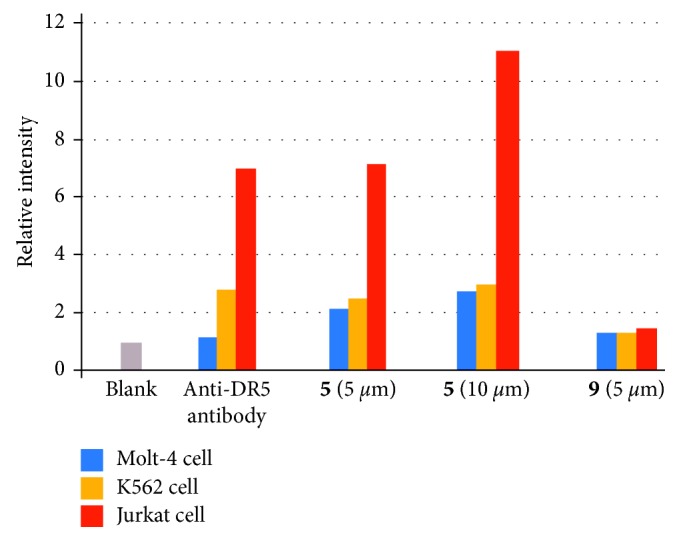
Summary of flow cytometry assay of DR5 expression of Molt-4 cells (blue bar), K562 cells (orange bar), and Jurkat cells (red bar). The cells were stained with anti-DR5 antibody (15 *µ*g/mL) at 4°C for 15 min. **5** (5/10 *µ*M) and **9** (5 *µ*M) at 37°C for 1 h. The relative intensity is the ratio of the geometric mean values of luminescence intensity to the blank.

To assess the affinity of IPH to DR5 expressed on cancer cell membrane, we carried out cell staining of different types of cancer cell lines that express different levels of DR5. DR5 expression of Jurkat cells, K562 cells, and Molt-4 cells was evaluated by staining with anti-DR5 antibody and flow cytometer analysis. It was found that Jurkat cells express comparatively high level of DR5 than K562 cells and Molt-4 cells, as confirmed by staining with anti-DR5 antibody (the second left in Figure 13). These three cell lines were incubated with **5** (5 *µ*M and 10 *µ*M) at 37°C for 1 h and then analyzed by flow cytometer. Figure 13 shows that, Jurkat cells are highly stained by **5**, while K562 cells and Molt-4 cells are weakly stained. Luminescence microscopic observation of different types of cancer cell lines also show similar results (Figures S7(a)–S7(i) in Supplementary Materials) and Ir complex **9** having no peptide [72] (Figure 6) negligibly stains Jurkat cells (Figures S7(j)–S7(l) in Supplementary Materials). Together with the results of the aforementioned QCM experiments (Figure 2), these facts strongly suggest that the binding of **5** is dependent on the DR5 expression level of cancer cells.

**Figure 14:**
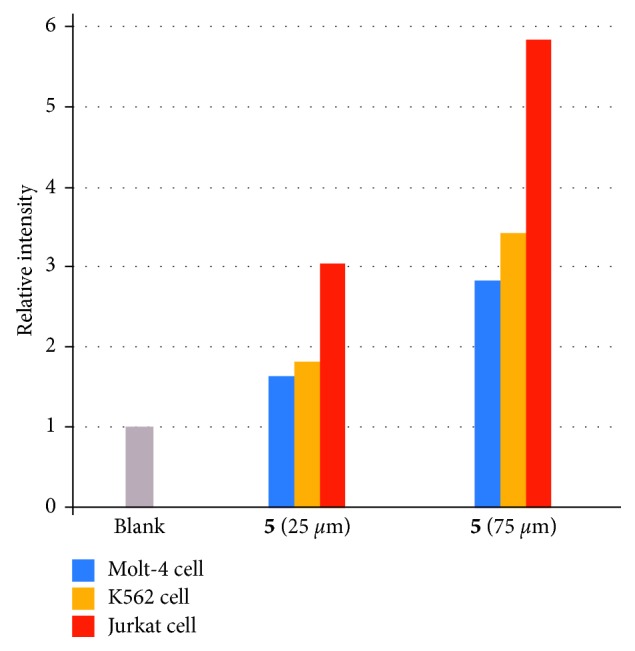
Summary of cell death assay (PI staining of dead cells) of Molt-4 cells (blue bar), K562 cells (orange bar), and Jurkat cells (red bar). The relative intensity is the ratio of the geometric mean values of the luminescence intensity to the blank.

Relationship between cell death inducing activity of IPH and DR5 expression of cancer cells was studied. Flow cytometry assay was conducted with Jurkat cells, K562 cells, and Molt-4 cells that express different level of DR5, as mentioned above. These three cell lines were incubated with **5** (25/75 *µ*M) at 37°C for 24 h, to which PI (30 *µ*M) was added to stain the dead cells for analysis by flow cytometer. As summarized in Figure 14, cell death induction of these three cell lines is parallel to expression level of DR5.

### 3.7. Mechanistic Studies of Cell Death Induced by IPH

The aforementioned results strongly suggest that **5** is able to detect Jurkat cells and induce their cell death via complexation with DR5. The stability of **5** in RPMI 1640 (MTG Free) medium (Incubation at 37°C for 24 h) was checked, as shown in Figure S8 in the Supplementary Materials. Since it is well established that DR5 initiates apoptosis signal after binding with TRAIL or other reported artificial TRAIL mimics, it was initially presumed that the cell death induced by **5** would be apoptosis. MTT assay of Jurkat cells with **5** was carried out in the presence of Z-VAD-FMK (15 *µ*M), which is a broad caspase inhibitor [76], necrostation-1 (30 *µ*M), which is a necroptosis inhibitor [77], and IM-54 (10 *µ*M), which is an inhibitor of oxidative stress induced necrosis [78]. However, these three drugs negligibly inhibited the cell death induced by **5** (Figures S9 and S10 in Supplementary Materials). On the other hand, TRAIL-induced cell death was inhibited by Z-VAD-FMK almost completely (Figures S9 and S10 in Supplementary Materials). In addition, the staining experiments of the dead Jurkat cells with annexin V-Cy3 [79, 80], which is a well-known reagent to detect apoptosis, disclosed that only few cells were stained by annexin V-Cy3. These facts have allowed us to conclude that it is unlikely that **5** induces apoptosis of Jurkat cells.

Aforementioned studies suggest that **5** internalizes into cells by DR5-mediated endocytosis. Therefore, **5** may interact with cytoplasmic organelles or interfere and/or activate any cellular events to induce cell death. In order to examine this mechanism, we evaluated the cell death in the presence of the inhibitors of several cellular events or metabolisms, channel blockers, or receptor antagonist, namely, carbonyl cyanide 3-chlorophenylhydrazone (CCCP) (a mitochondrial uncoupling reagent) [81, 82], amiloride (Na^+^ channel blocker, inhibitors of macropinocytosis) [83], chloroquine (inhibitors of autophagy) [84], bafilomycin A1 (an inhibitor of vacular ATPase (V-ATPase) [85], oligomycin (an inhibitor of the mitochondrial F1/F0-ATP synthase to cause ATP depletion) [86], 4-aminopyridine (K^+^ channel blocker), verapamil (L-type voltage-operated Ca^2+^ channel blocker) [87–89], nicardipine, (L-type voltage-operated Ca^2+^ channel blocker) [87–89], and quinidine, (Na^+^ and K^+^ channel blocker, antagonist of *α*-adrenergic receptors, and an inhibitor of the mitochondrial uptake of Ca^2+^) [90–94], according to our previous studies [72, 73]. Jurkat cells were incubated with these inhibitors at their recommended concentrations (Figure S11 in Supplementary Materials) at 37°C for 1 h, to which **5** was added and incubated again at the same temperature for 24 h. The cells were then washed with PBS buffer and stained with propidium iodide (PI) for microscopic observation. Interestingly, it was found that only voltage-operated Ca^2+^ channel blocker (nicardipine and verapamil) considerably inhibited cell death induced by **5**. Therefore, we conclude that the cell death induced by **5** can be considered as necrosis-type cell death via Ca^2+^-mediated intracellular signaling pathway.

### 3.8. Detection of Jurkat Cells in Bovine Blood

**Figure 15:**
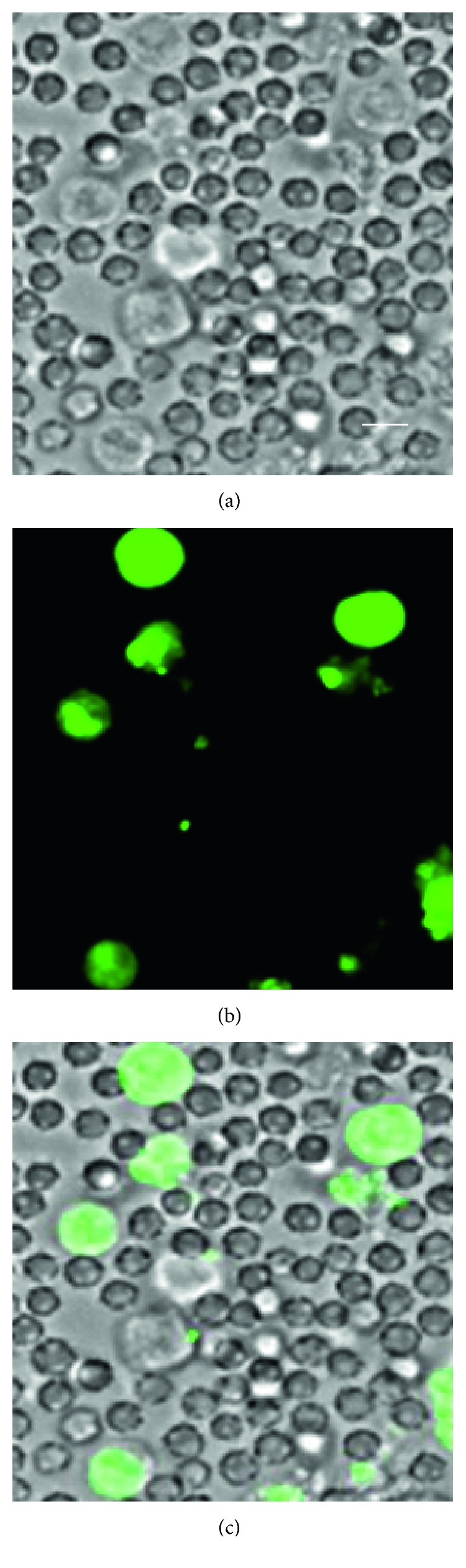
Luminescence microscopy images (×40) (Biorevo, BZ-9000, Keyence) of Jurkat cells spiked in bovine blood in presence of **5**. (a–c) Jurkat cells spiked in bovine blood after incubation with **5** (10 *µ*M) at 37°C for 6 h: (a) bright field image, (b) emission image, and (c) overlay image of (a) and (b). Scale bar (white) = 10 *µ*m.

The detection of cancer cells by use of IPHs was carried out. Jurkat cells (10 × 10^5^ cells) were suspended in bovine blood (100 *µ*L) and then incubated with **5** (10 *µ*M) in medium (RPMI 1640) at 37°C for 6 h, resulting in the successful detection of Jurkat cells in bovine blood, as shown in Figure 15.

## 4. Conclusions

In this study, we report on the design and synthesis of Ir complex-peptide hybrids (**4**–**6**) consisted of *C*_3_-symmetric tris-cyclometalated Ir complexes equipped with DR5 binding cyclic peptides. Among these complexes, **5** and **6** were found to be cytotoxic against Jurkat cells. Studies demonstrate that **5** binds with DR5 on the cell membrane (by 27 MHz QCM and costaining experiments with anti-DR5 antibody), and their complex is internalized into the cytoplasm by DR5-mediated endocytosis. Our previous Ir complexes **2**–**3** having basic peptides such as KKGG sequence induce Jurkat cell death in a few hours and exhibit strong emission from dead cells. In contrast, the IPHs reported in this study (**5** and **6**) detect Jurkat cells first and then induce their death very slowly (after 16 to 24 h). Moreover, DR5 is transferred and/or reproduced on the cell surface after additional incubation. Jurkat cells can be detected even in bovine blood which may offer an easy and convenient method for cancer diagnosis. To the best of our knowledge, **5** is the first example of the artificial compound that achieve imaging and cell death induction of cancer cells associated with DR5, due to its moderate cytoxicity and slow cell death induction property.

The aforementioned results postulate that IPHs can be good candidates to study death receptor biology, cancer cell imaging, induction of cancer cell death, and understanding of the mechanism of cell death mediated by death receptor. Additionally, IPHs induce slow cell death, which may provide new approaches in the treatment of cancer and related diseases. Improvement of the anticancer activity of IPHs and attempts at controlling cell death types are now in progress.

## Figures and Tables

**Table 1:** Photophysical properties of Ir complexes **2c**, **4**, **5,** and **6** in degassed DMSO at 25°C ([Ir complex] = 10 *µ*M, excitation at 366 nm).

Compound	*λ*_max_ (absorption) (nm)	*λ*_max_ (emission) (nm)	Φ^a^	*τ* ^b^ (*µ*s)
**2c**	280, 362	509	0.55	1.7
**4**	285, 363	505	0.39	1.1
**5**	285, 361	506	0.33	1.3
**6**	286, 360	506	0.36	1.2

^a^Quinine sulfate in 0.1 M H_2_SO_4_ (Φ = 0.55) was used as a reference. ^b^Lifetime of luminescence emission.

**Table 2:** Complexation constants of IPHs (assuming 1 : 1 complexation).

Analyte	*K*_app_ (M^−1^)	*K*_d_
TRAIL	(2.3 ± 0.05) × 10^8^	4.3 ± 0.1 nm
**5**	(3.8 ± 0.1) × 10^5^	2.7 ± 0.1 *μ*M
**6**	(4.0 ± 0.2)10^5^	2.5 ± 0.1 *μ*M
**9**	<10^4^	>100 *μ*M
**2c**	<10^4^	>100 *μ*M

## Abbreviations

DIC: *N*,*N*′-diisopropylcarbodiimide
DIEA: *N*,*N*-diisopropylethylamine
DMF: *N*,*N*-dimethylformamide
EDC: 1-Ethyl-3-(3-dimethylaminopropyl)carbodiimide
HOBt: 1-Hydroxybenzotriazole
NHS: *N*-hydroxy succinimide
PyBOP: Benzotriazol-1-yl-oxytripyrrolidinophosphonium hexafluorophosphate
TFA: Trifluoroacetic acid
THF: Tetrahydrofuran
TIPS: Triisopropylsilane
TMSCl: Trimethylsilyl chloride.

## Supplementary Materials

Supplementary Materials Figure S1: the results of MTT assay. Figure S2: microscopic images of competitive staining. Figure S3: flow cytometry assay results of competitive staining. Figures S4–S6: flow cytometry assay results of costaining Figure S7: microscopic images of Jurkat, K562, and Molt-4 cells staining. Figure S8: HPLC charts of **5** after incubation in RPMI 1640 medium. Figures S9 and S10: the results of MTT assay in presence of inhibitors. Figure S11: microscopic images of staining in presence of inhibitors. Figure S12–13: ^1^H NMR and ESI mass charts of NHS ester of Ir complex **7**. Figures S14–S15: ^1^H NMR and ESI mass charts of Ir complex **8**. Figures S16 and S17: ^1^H NMR and ESI mass charts of NHS ester of Ir complex **8**. Figures S18 and S19: ^1^H NMR and ESI mass charts of Ir complex **9**. Figures S20–S22: HPLC, ^1^H NMR, and ESI mass charts of **CP1**. Figures S23–S25: HPLC, ^1^H NMR, and ESI mass charts of **CP2**. Figures S26–S28: HPLC, ^1^H NMR, and ESI mass charts of **CP3**. Figure S29–S31: HPLC, ^1^H NMR, and ESI mass charts of Ir complex **4**. Figures S32–S34: HPLC, ^1^H NMR, and ESI mass charts of Ir complex **5**. Figures S35–S37: HPLC, ^1^H NMR, and ESI mass charts of Ir complex **6**.
