# Supplementary material for: Luminescent Iridium Complex-Peptide Hybrids (IPHs) for Therapeutics of Cancer: Design and Synthesis of IPHs for Detection of Cancer Cells and Induction of Their Necrosis-Type Cell Death
Source: Bioinorg Chem Appl. 2018 Aug 1;2018:7578965. doi: 10.1155/2018/7578965 (PMC6092981; doi:10.1155/2018/7578965)
Supplement: Supplementary Materials — Figure S1: the results of MTT assay. Figure S2: microscopic images of competitive staining. Figure S3: flow cytometry assay results of competitive staining. Figures S4–S6: flow cytometry assay results of costaining Figure S7: microscopic images of Jurkat, K562, and Molt-4 cells staining. Figure S8: HPLC charts of 5 after incubation in RPMI 1640 medium. Figures S9 and S10: the results of MTT assay in presence of inhibitors. Figure S11: microscopic images of staining in presence of inhibitors. Figure S12–13: 1H NMR and ESI mass charts of NHS ester of Ir complex 7. Figures S14–S15: 1H NMR and ESI mass charts of Ir complex 8. Figures S16 and S17: 1H NMR and ESI mass charts of NHS ester of Ir complex 8. Figures S18 and S19: 1H NMR and ESI mass charts of Ir complex 9. Figures S20–S22: HPLC, 1H NMR, and ESI mass charts of CP1. Figures S23–S25: HPLC, 1H NMR, and ESI mass charts of CP2. Figures S26–S28: HPLC, 1H NMR, and ESI mass charts of CP3. Figure S29–S31: HPLC, 1H NMR, and ESI mass charts of Ir complex 4. Figures S32–S34: HPLC, 1H NMR, and ESI mass charts of Ir complex 5. Figures S35–S37: HPLC, 1H NMR, and ESI mass charts of Ir complex 6. [file 7578965.f1.pdf]

Supplementary Materials for

Luminescent Iridium Complex-Peptide Hybrids (IPHs) for Therapeutics of Cancer: Design and Synthesis of IPHs for Detection of Cancer Cells and Induction of Their Necrosis-type Cell Death

Abdullah-Al Masum,[a] *Yosuke Hisamatsu,*[a] *Kenta Yokoi,*[a] and *Shin Aoki*[*,a,b]

[a] Faculty of Pharmaceutical Sciences, Tokyo University of Science,

2641 Yamazaki, Noda, Chiba 278-8510, Japan,

[b] Imaging Frontier Center, Tokyo University of Science

2641 Yamazaki, Noda, Chiba 278-8510, Japan

*Corresponding author: E-mail, shinaoki@rs.noda.tus.ac.jp

Contents

Figure S1. The results of MTT assay……………………..…….….………..………..…S3

Figure S2. Microscopic images of competitive staining ……..…..……………...………..……S3

Figure S3. Flow cytometry assay results of competitive staining ………………….…..………S4

Figure S4-6. Flow cytometry assay results of co-staining ……….…………………………S4-S6

Figure S7. Microscopic images of Jurkat, K562 and Molt-4 cells staining ……..……..………S7

Figure S8. HPLC charts of **5** after incubation in RPMI 1640 medium ……………..….……....S8

Figure S9-10. The results of MTT assay in presence of inhibitors …….………….……….…S9-10

Figure S11. Microscopic images of staining in presence of inhibitors ….…..………….……….S11

Figure S12-13. ^{1}H NMR & ESI mass charts of NHS ester of Ir Complex **7** ….....………..….S12

Figure S14-15. ^{1}H NMR & ESI mass charts of Ir Complex **8** ….……………...……….…….S13

Figure S16-17. ^{1}H NMR & ESI mass charts of NHS ester of Ir Complex **8** …...………..…...S14

Figure S18-19. ^{1}H NMR & ESI mass charts of Ir Complex **9** ……..…………………..……..S15

Figure S20-22. HPLC, ^{1}H NMR & ESI mass charts of **CP1** …...……………………..….S16-17

Figure S23-25. HPLC, ^{1}H NMR & ESI mass charts of **CP2** ……..……………………...….S17-18

Figure S26-28. HPLC, ^{1}H NMR & ESI mass charts of **CP3** ……...…………………..….S19-20

Figure S29-31. HPLC, ^{1}H NMR & ESI mass charts of Ir complex **4** …………......…….S20-21

Figure S32-34. HPLC, ^{1}H NMR & ESI mass charts of Ir complex **5** …………...……….S22-23

Figure S35-37. HPLC, ^{1}H NMR & ESI mass charts of Ir complex **6** ……………...…….S23-24

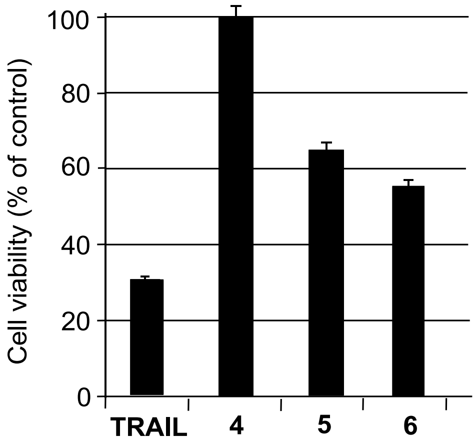

Figure S1. The results of MTT assay: cell viability of Jurkat cells (% of control at [Ir complex] = 0 μM) in presence of TRAIL (150 ng/mL), **4**, **5** and **6** (75 μM) after incubation at 37 ^{o}C for 16 h.

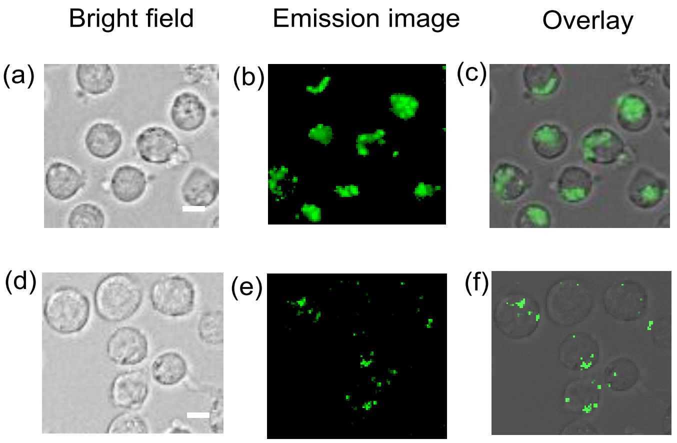

Figure S2. The results of competitive staining of Jurkat cells with **5** and **CP1** (luminescence microscopy images on Biorevo, BZ-9000, Keyence, x 40). (a-c) Jurkat cells were incubatied with **5** (10 µM) at 37˚C for 1 h. (d-f) Jurkat cells were incubated with **CP1** (100 μM) at 37˚C for 1 h and then with **5** (10 μM) at 37˚C for 1 h. Scale bar (white) = 10 μm.

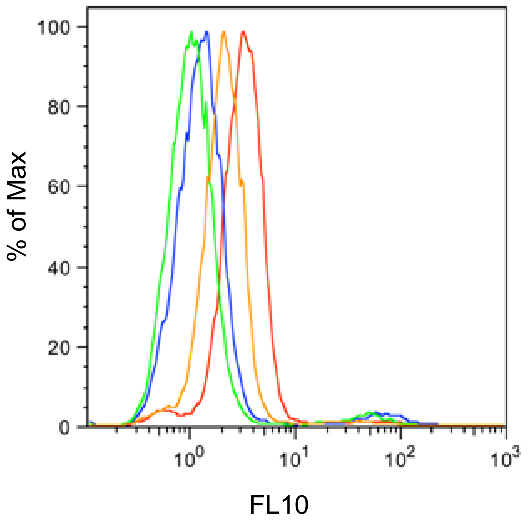

Figure S3. Flow cytometry analysis of competitive staining of Jurkat cells with **5** and **CP1**. The red histogram corresponds to Jurkat cells treated with **5** (5 μM) at 37 °C for 1 h**,** the orange histogram corresponds to Jurkat cells treated with **CP1** (100 μM) at 37 °C for 1 h and then **5** (5 μM) at 37 °C for 1 h, the blue histogram corresponds to Jurkat cells treated with **5** (2 μM) at 37 °C for 1 h and the green histogram corresponds to the cells treated with **CP1** (100 μM) at 37 °C for 1 h and then **5** (2 μM) at 37 °C for 1 h.

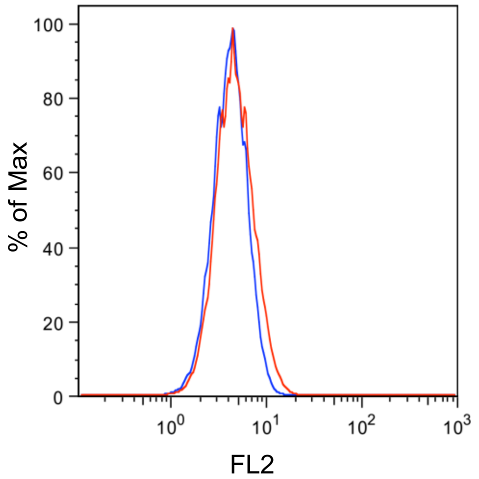

Figure S4. Flow cytometry analysis of co-staining assay of anti-DR5 antibody and **5.** The blue histogram corresponds to the cells treated with anti-DR5 antibody (15 μg/mL) at 4˚C for 15 min, and the red histogram corresponds to the cells treated with anti-DR5 antibody (15 μg/mL) at 4˚C for 15 min and then with **5 (**5 μM) at 37˚C for 1 h.

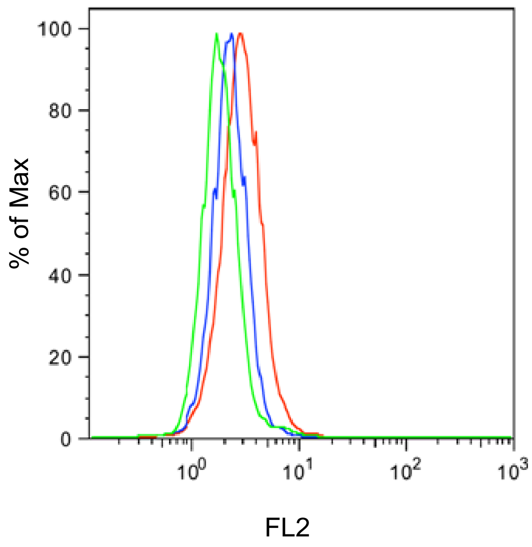

Figure S5. Flow cytometry analysis of co-staining of Jurkat cells with **5** and anti-DR5 antibody. The red histogram corresponds to the cells treated with only anti-DR5 antibody (15 μg/mL) at 4 °C for 15 min (Method a of Chart 5 in text), the blue histogram corresponds to the cells treated with **5** (5 μM) at 37 °C for 1 h and then anti-DR5 antibody (15 μg/mL) at 4 °C for 15 min (Method c of Chart 5 in text), and the green histogram corresponds to the cells treated with **5** (10 μM) at 37 °C for 1 h and then anti-DR5 antibody (15 μg/mL) at 4 °C for 15 min (Method c of Chart 5 in text).

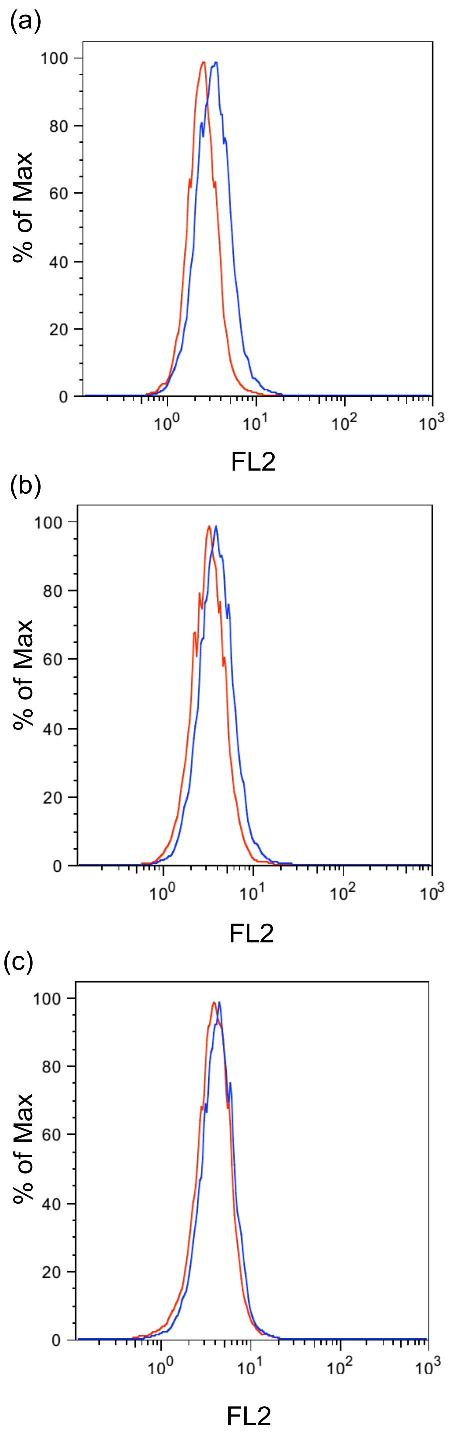

Figure S6. Flow cytometry analysis of Jurkat cells incubated with **5** (5 μM) and anti-DR5 antibody (15 μg/mL). Red histograms correspond to the cells treated with **5** and anti-DR5 antibody, blue histograms correspond to the cells treated with only anti-DR5 antibody. (a) after 1 h incubation with **5** (Method c in Chart 5 (b) after 1 h incubation with **5** and then again 1 h incubation in fresh medium (Method d in Chart 5). (c) after 1 h incubation with **5** and then again 6 h incubation in fresh medium (Method d in Chart 5).

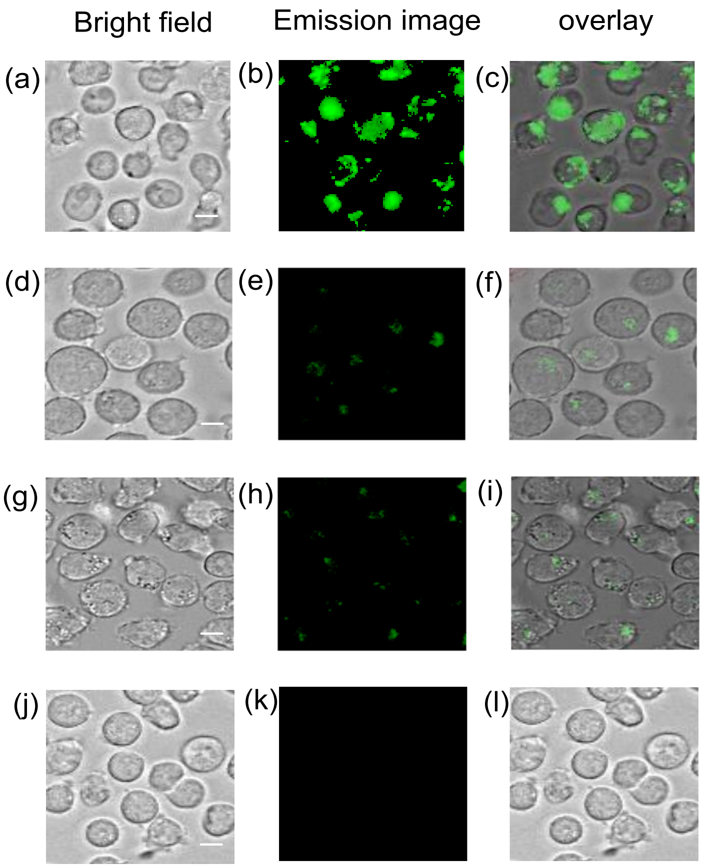

Figure S7. Luminescence microscopy images (Biorevo, BZ-9000, Keyence) of Jurkat cells, K562 cells and Molt-4 cells (x 40) stained with **5.** (a-c) Jurkat cells were incubated with **5** (10 μM) at 37˚C for 1 h. (d-f) K562 cells were incubated with **5** (10 μM) at 37˚C for 1 h. (g-i) Molt-4 cells were incubated with **5** (10 μM) at 37˚C for 1 h. (j-l) Jurkat cells were incubated with **9** (5 μM) at 37˚C for 1 h. Scale bar (white) = 10 μm.

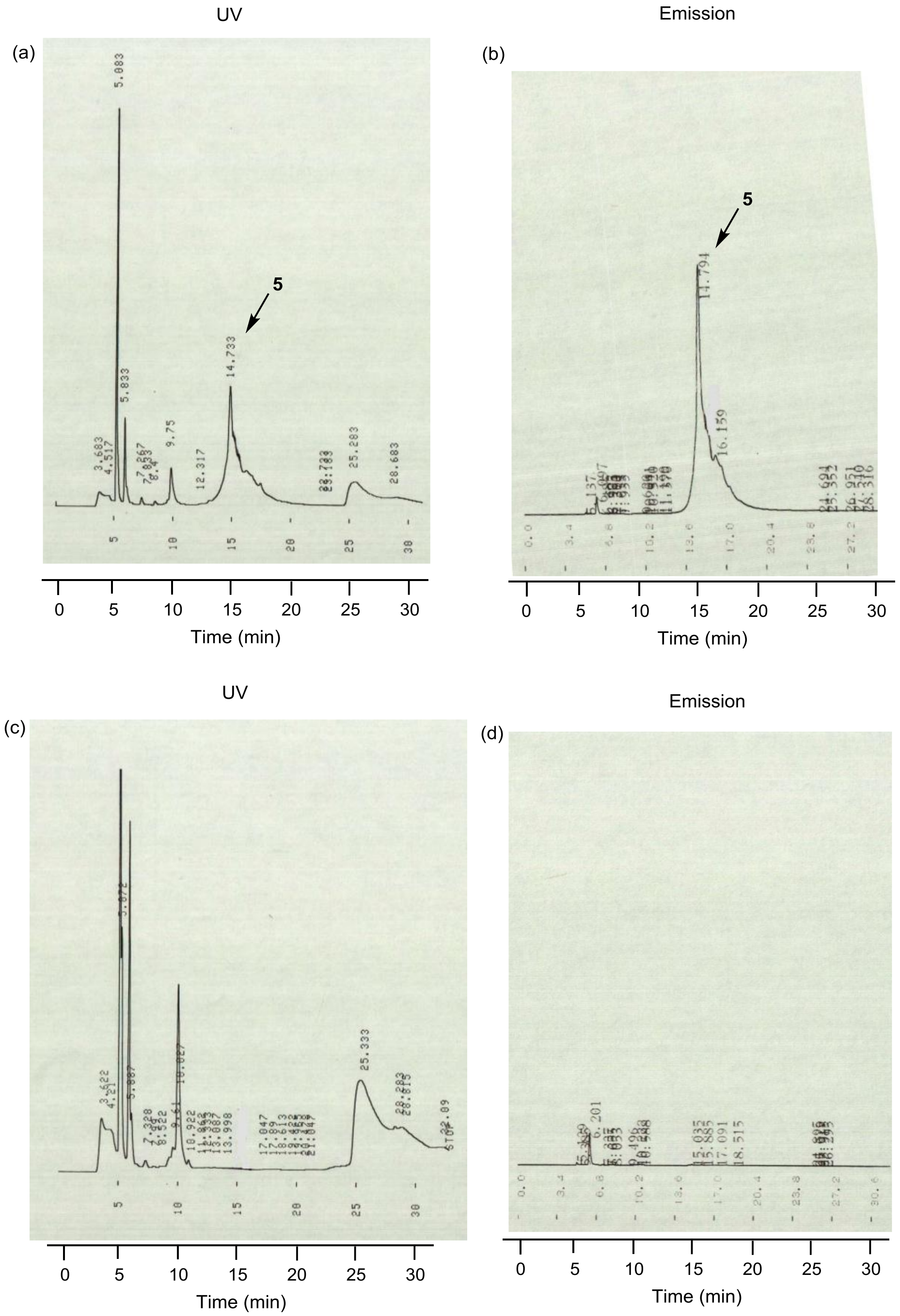

Figure S8. HPLC charts of (a,b) **5** after incubation at 37 ^{o}C in RPMI 1640 (MTG free) medium for 24 h. (c,d) only RPMI 1640 (MTG free) medium (without **5**) after incubation at 37 ^{o}C for 24 h. (Senshu Pak PEGASIL ODS 4.6 ϕ X 250 mm UV: 254 nm, Excitation: 366 nm, Emission: 520 nm, flow rate 1.0 mL/min with continuous gradient elution (20-50% solvent B, 0-30 min, linear) with solvent A (0.1% TFA in H_2O) and solvent B (0.1% TFA in MeCN).

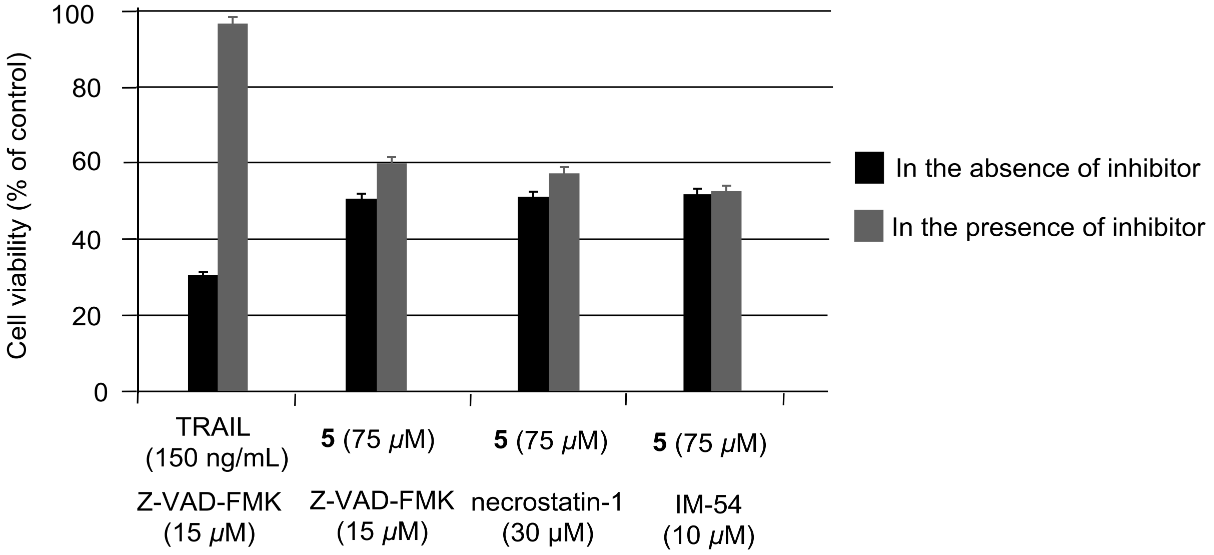

Figure S9. The results of MTT assay of TRAIL (150 ng/mL) and **5** (75 μM) in the presence and the absence of caspase inhibitor (Z-VAD-FMK), necroptosis inhibitor (Necrostatin-1) and oxidative stress induced necrosis inhibitor (IM-54): cell viability of Jurkat cells (% of control) after incubation at 37 ^{o}C for 24 h.

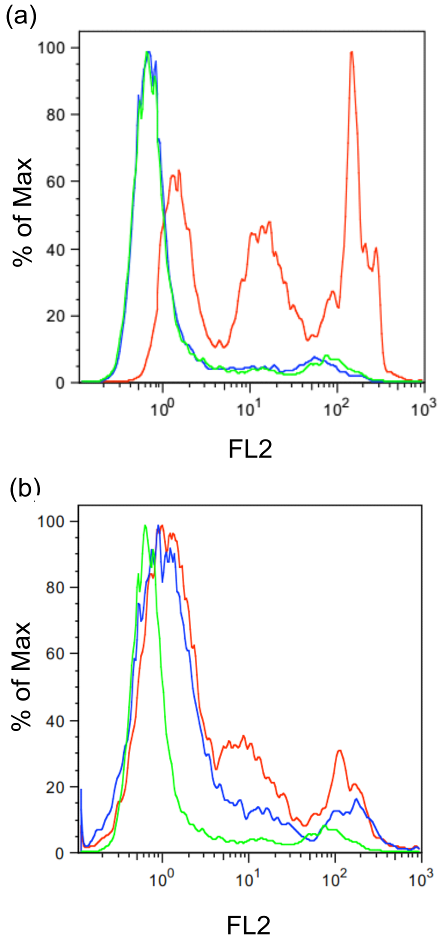

Figure S10. The results of flow cytometry assay of (a) TRAIL (150 ng/mL) and (b) **5** (75 μM). The red histograms correspond to the Jurkat cells treated with TRAIL (150 ng/mL) or **5** (75 μM) at 37 °C for 24 h and then with PI (30 μM) at room temperature for 10-15 min. The blue histograms correspond to the Jurkat cells treated with caspase inhibitor (Z-VAD-FMK) (15 μM) at 37 °C for 1h and then TRAIL (150 ng/mL) or **5** (75 μM) at 37 °C for 24 h followed by the treatment with PI (30 μM) at room temperature for 10-15 min. The green histograms correspond to the Jurkat cells not treated with TRAIL or **5**.

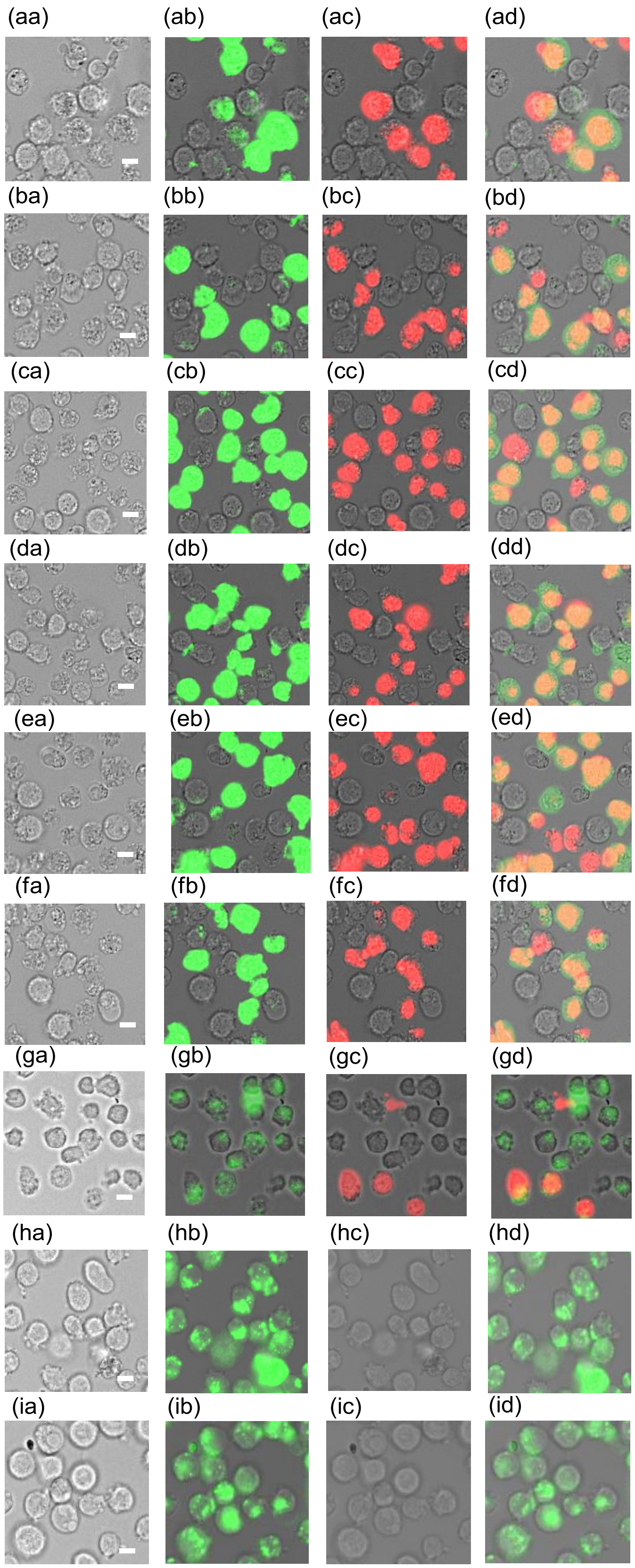

Figure S11. Luminescent microscopy images (Biorevo, BZ-9000, Keyence) of Jurkat cells (x 40) incubated with **5** (35 μM) in presence of (aa-ad) 4-aminopyridine (10 μM), (ba-bd) amiloride (100 μM), (ca-cd) bafilomycin A1 (10 μM), (da-dd) CCCP (40 μM), (ea-ed) chloroquinine (10 μM), (fa-fd)

phentolamine (10 μM), (ga-gd) quinidine (100 μM), (ha-hd) nicardipine (50 μM), and (ia-id) verapamil (20 μM) at 37 ^{o}C for 24 h. Scale bar (white) = 10 μm.

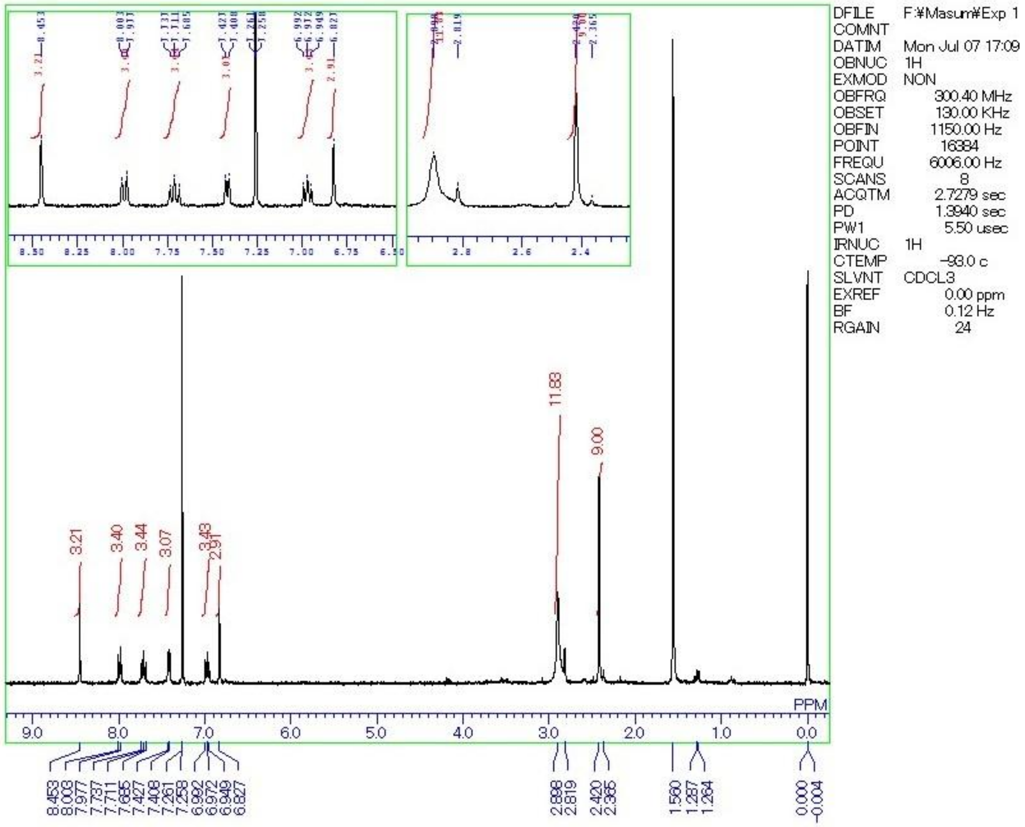

Figure S12: ^{1}H NMR chart of NHS ester of Ir complex **7**

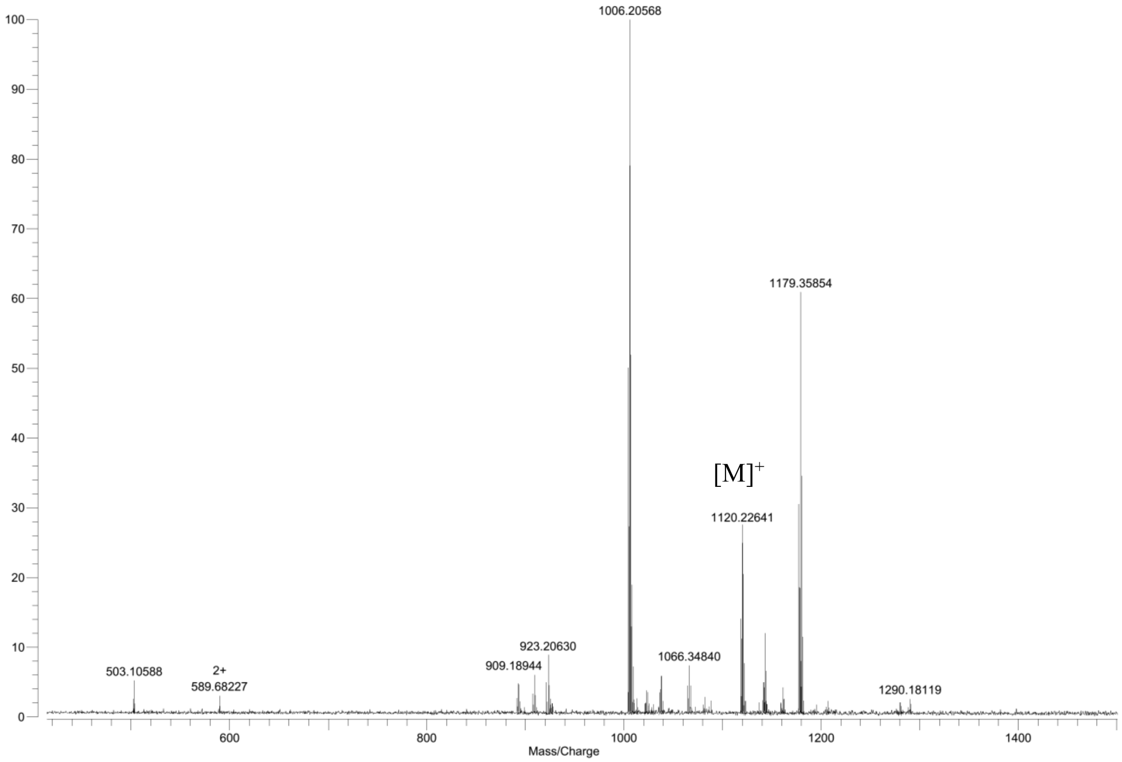

Figure S13: ESI mass chart of NHS ester of Ir complex **7**

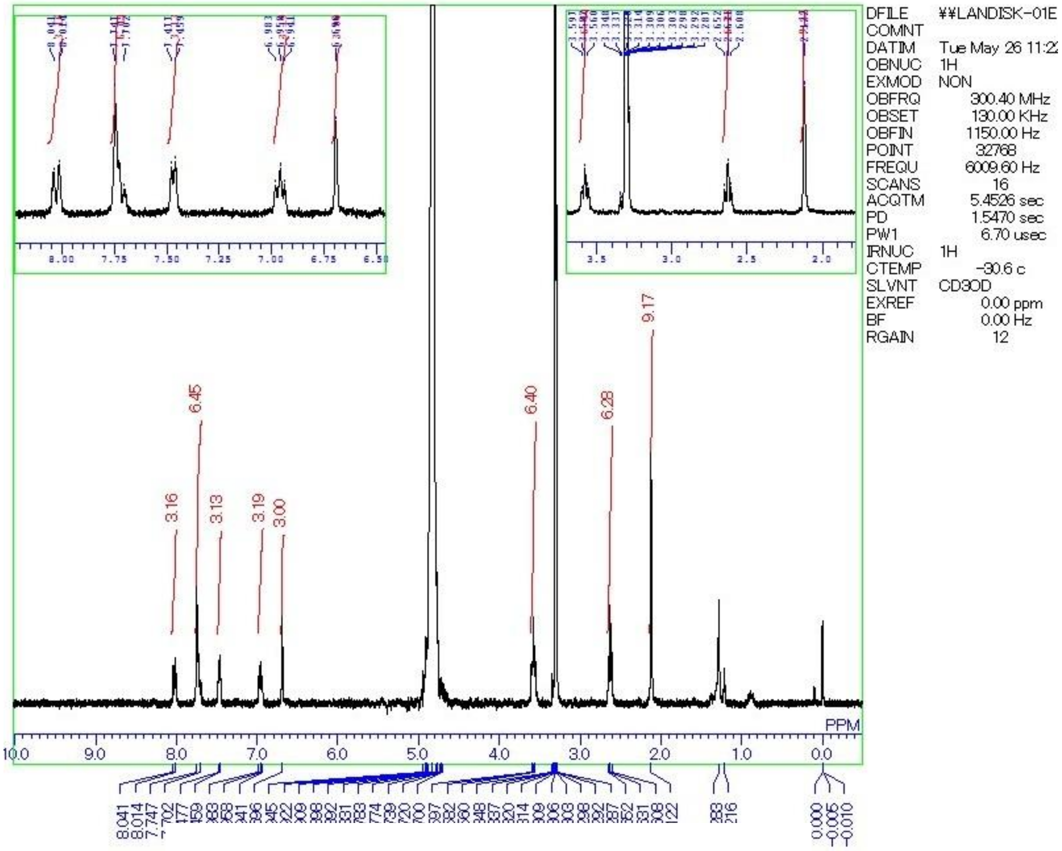

Figure S14: ^{1}H NMR chart of Ir complex **8**

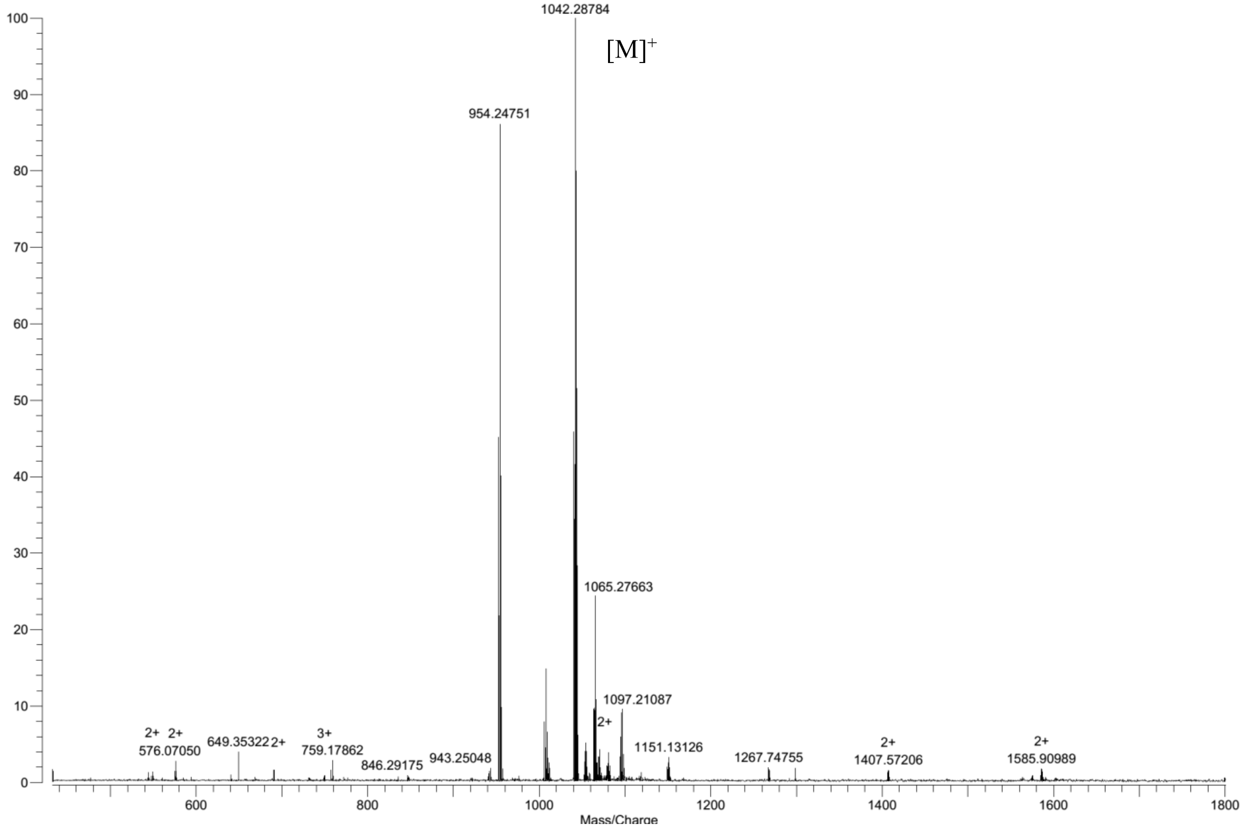

Figure S15: ESI mass chart of Ir complex **8**

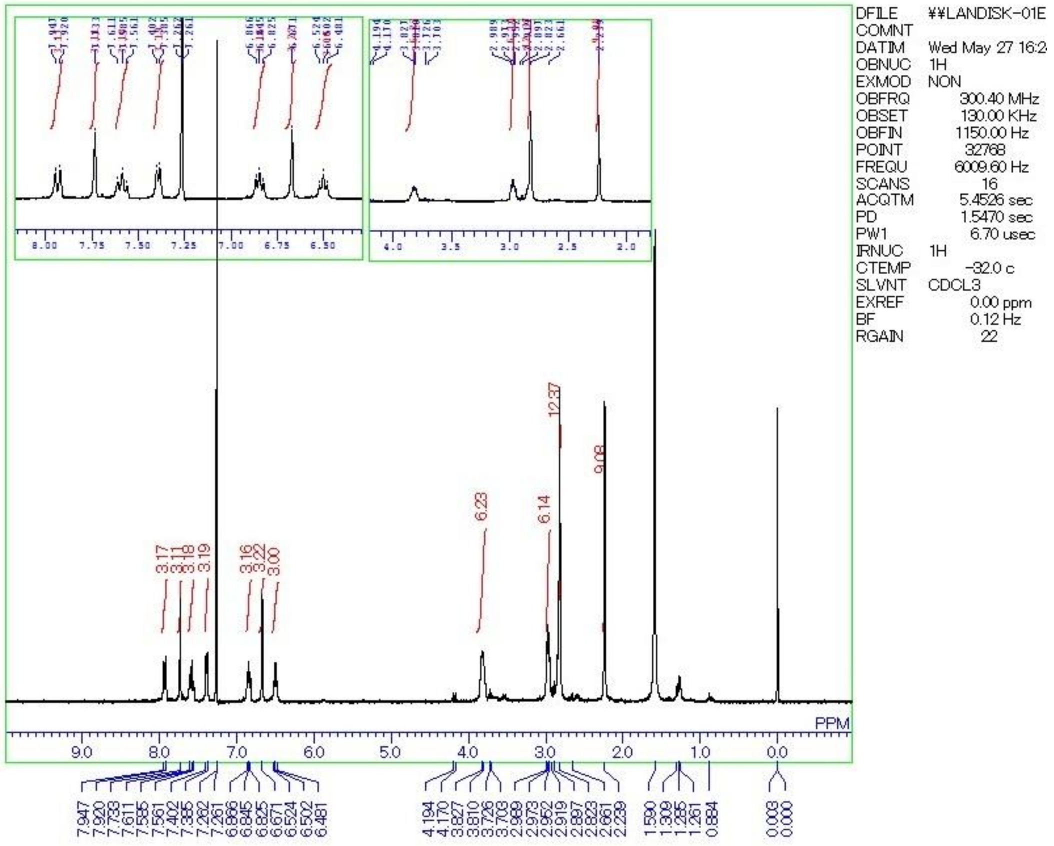

Figure S16: ^{1}H NMR chart of NHS ester of Ir complex **8**

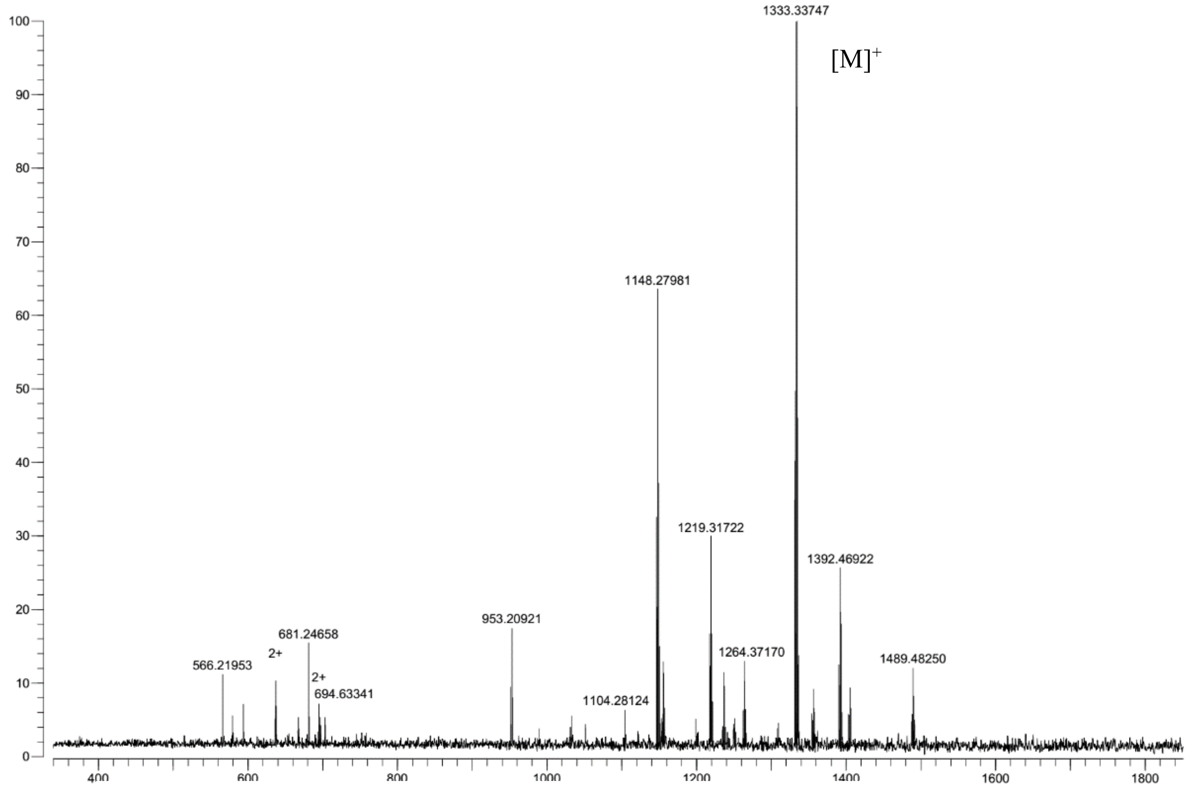

Figure S17: ESI mass chart of NHS ester of Ir complex **8**

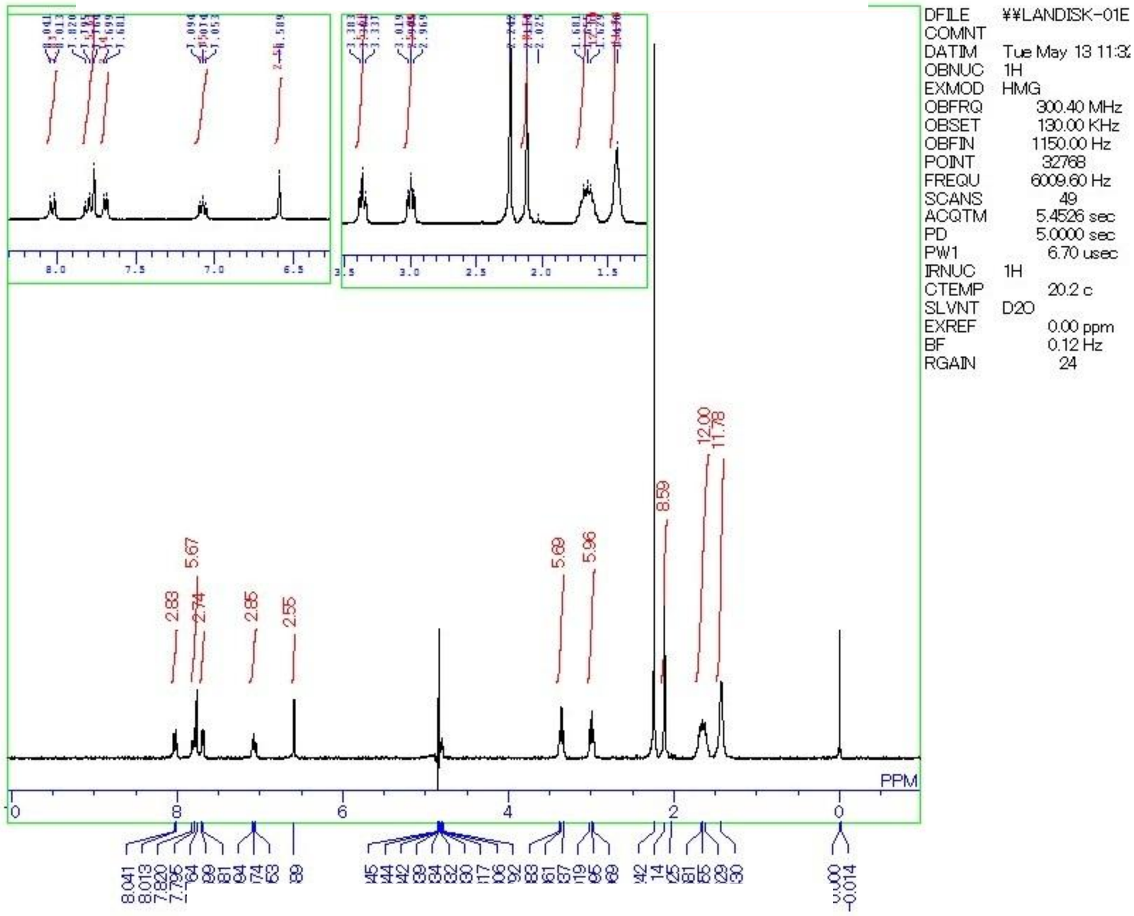

Figure S18: ^{1}H NMR chart of Ir complex **9**

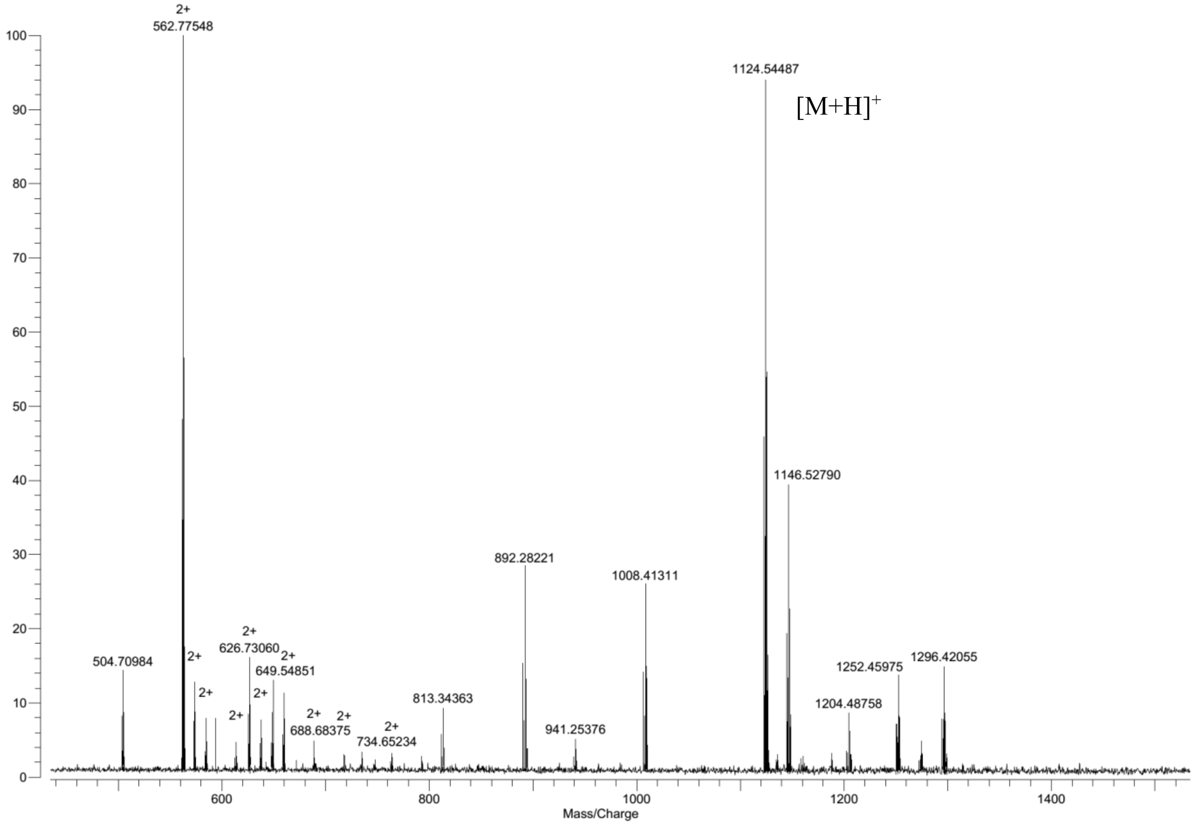

Figure S19: ESI mass chart of Ir complex **9**

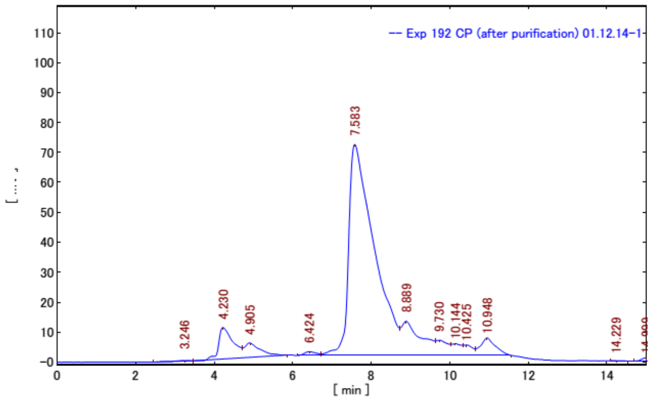

Senshu Pak PEGASIL ODS 4.6 ϕ X 250 mm UV: 280 nm, flow rate 1.0 mL/min with continuous gradient elution (20-50% solvent B, 0-30 min, linear) with solvent A (0.1% TFA in H_2O) and solvent B (0.1% TFA in MeCN).

Figure S20: HPLC analysis of **CP1**

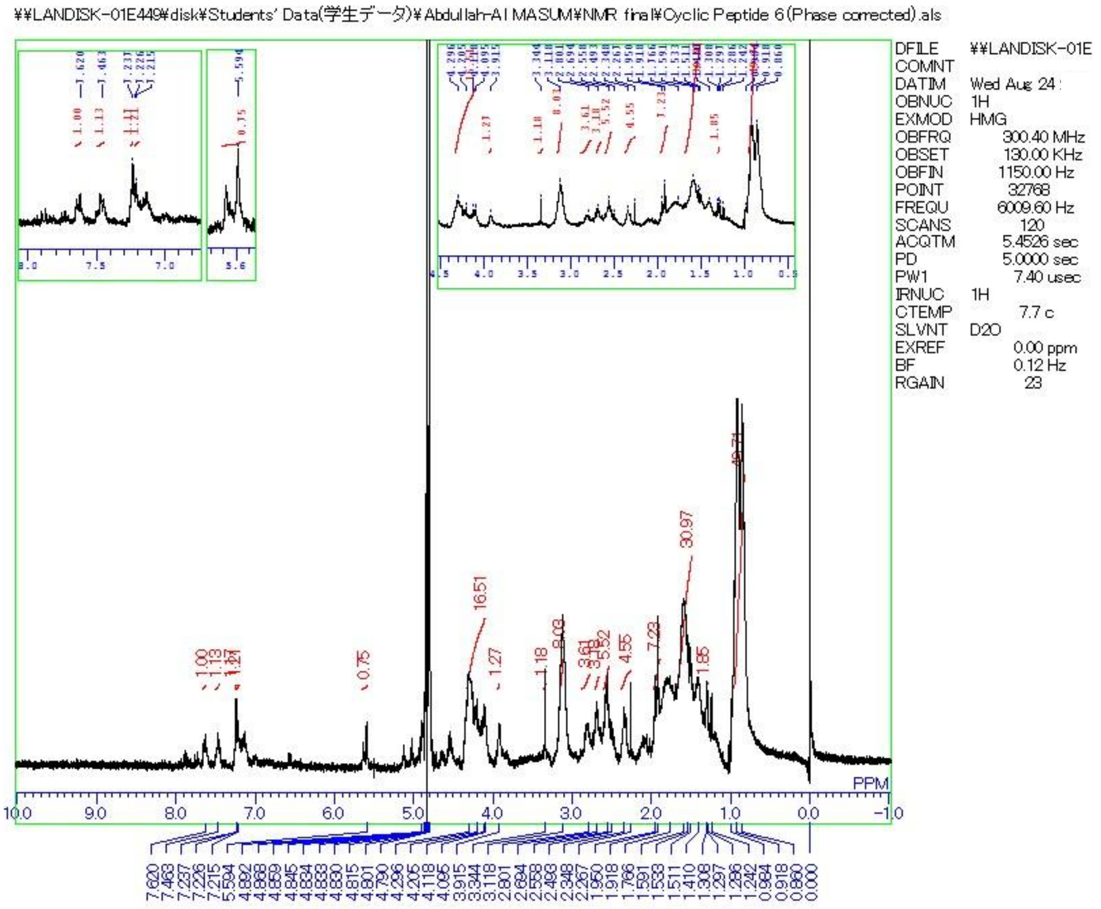

Figure S21: ^{1}H NMR chart of **CP1**

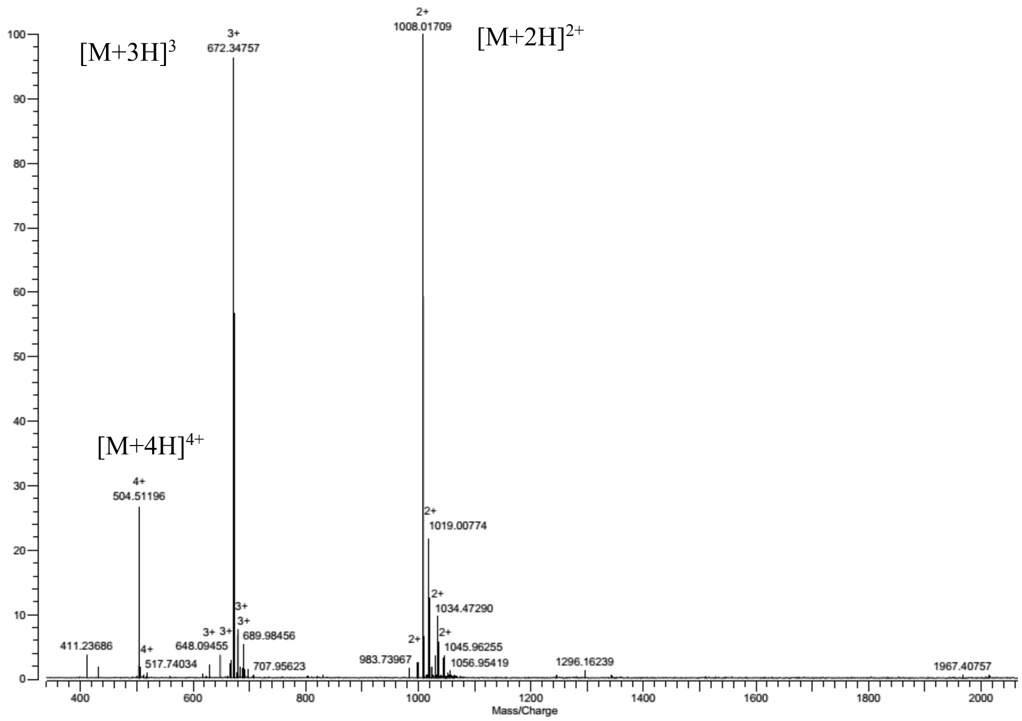

Figure S22: ESI Mass chart of **CP1**

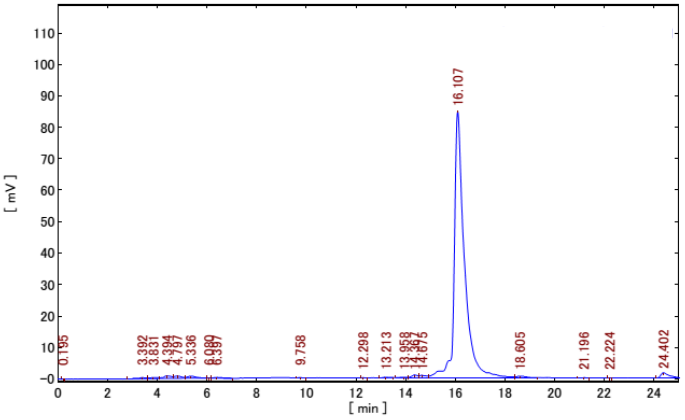

Senshu Pak PEGASIL ODS 4.6 ϕ X 250 mm

UV: 280 nm, flow rate 1.0 mL/min with continuous gradient elution (10-40% solvent B, 0-30 min, linear) with solvent A (0.1% TFA in H_2O) and solvent B (0.1% TFA in MeCN).

Figure S23: HPLC analysis of **CP2**

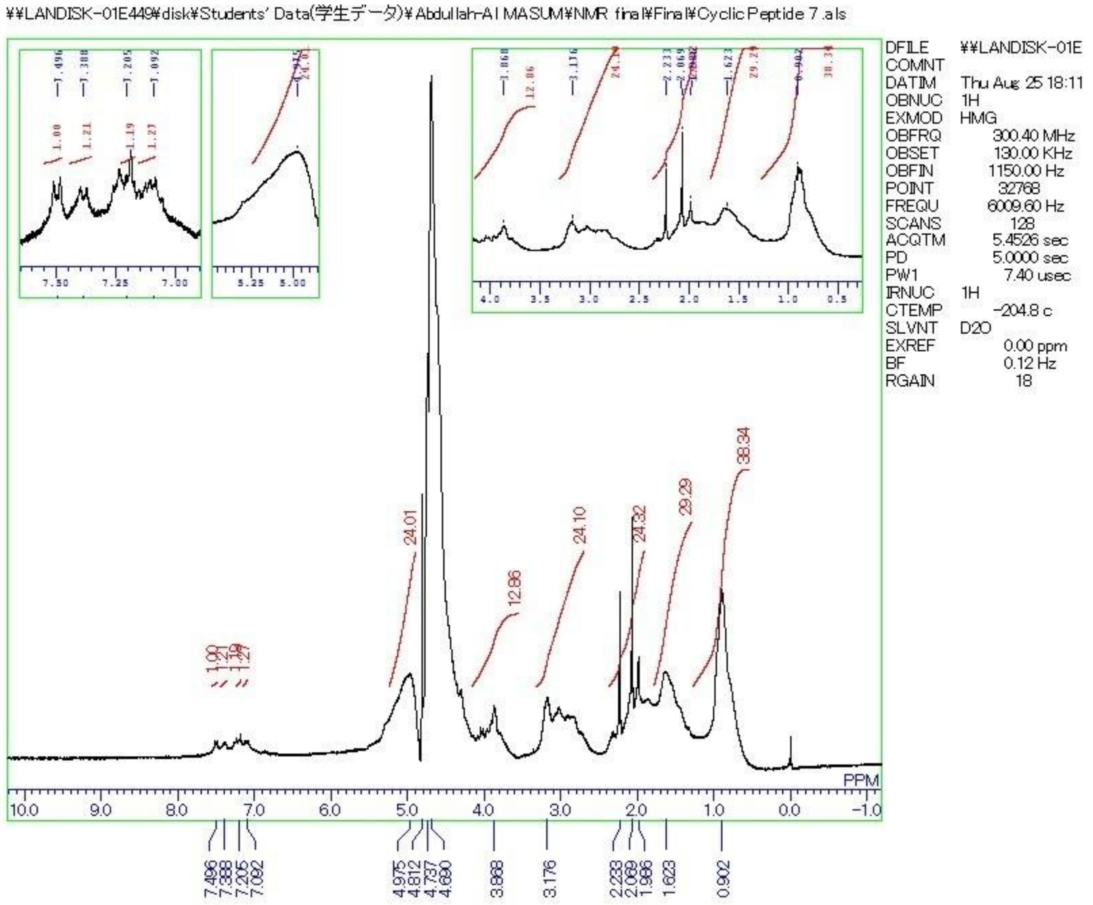

Figure S24: ^{1}H NMR chart of **CP2**

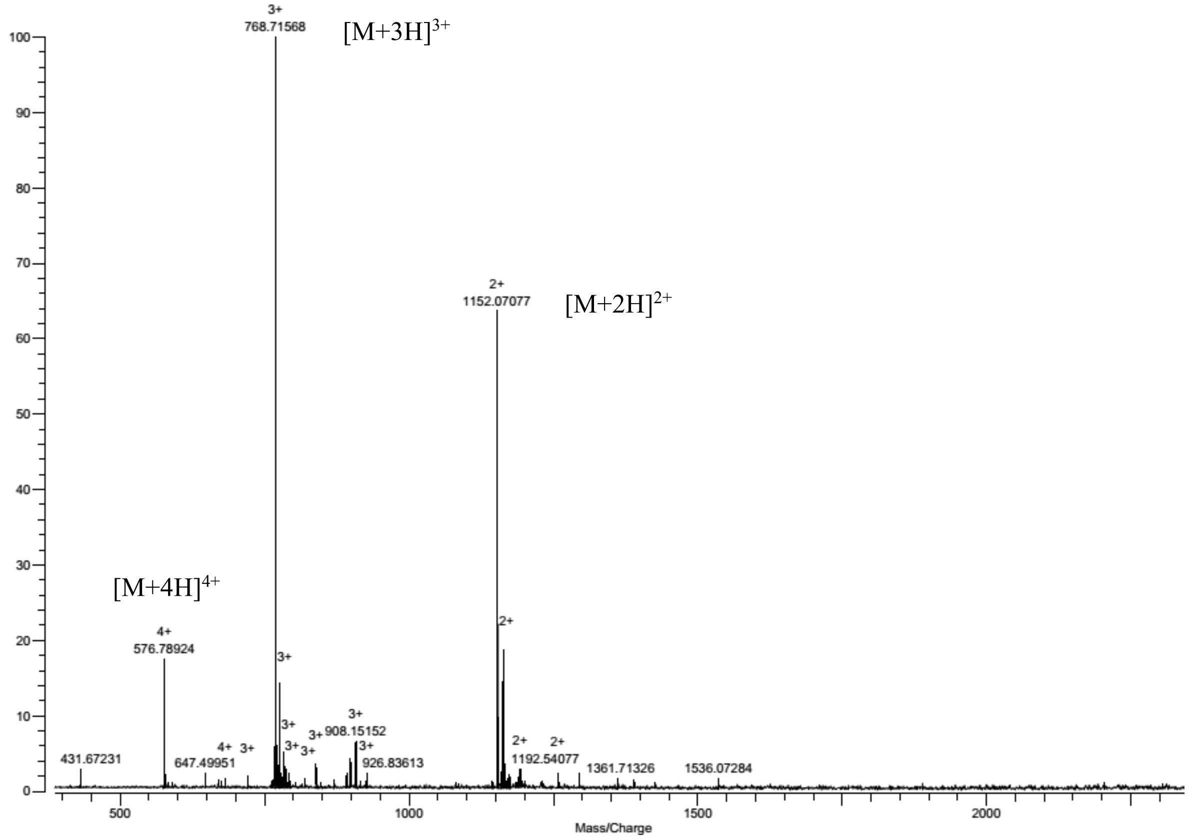

Figure S25: ESI Mass chart of **CP2**

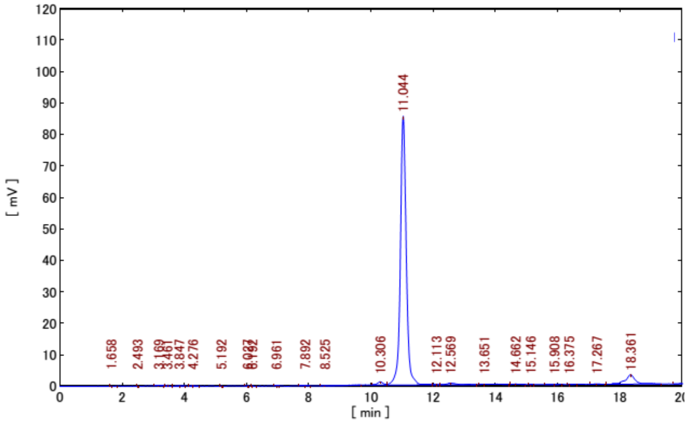

Senshu Pak PEGASIL ODS 4.6 ϕ X 250 mm UV: 280 nm, flow rate 1.0 mL/min with continuous gradient elution (10-40% solvent B, 0-30 min, linear) with solvent A (0.1% TFA in H_2O) and solvent B (0.1% TFA in MeCN).

Figure S26: HPLC analysis of **CP3**

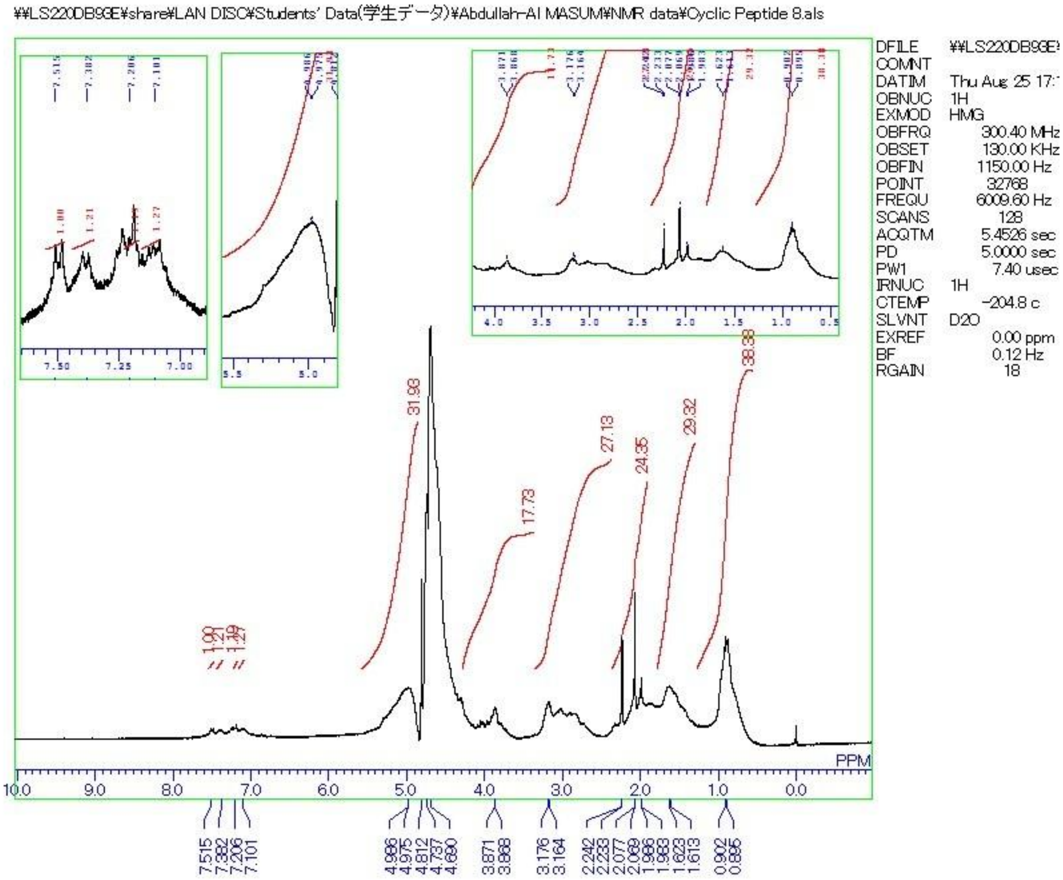

Figure S27: ^{1}H NMR chart of **CP3**

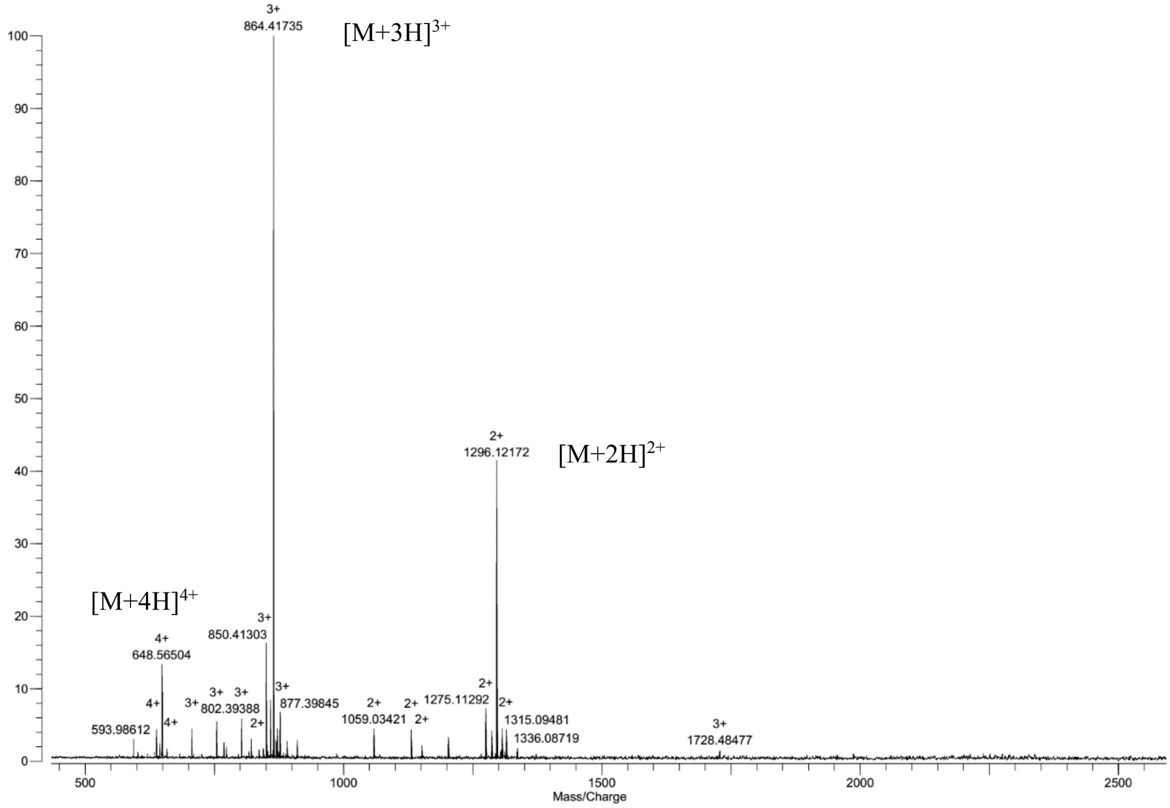

Figure S28: ESI Mass chart of **CP3**

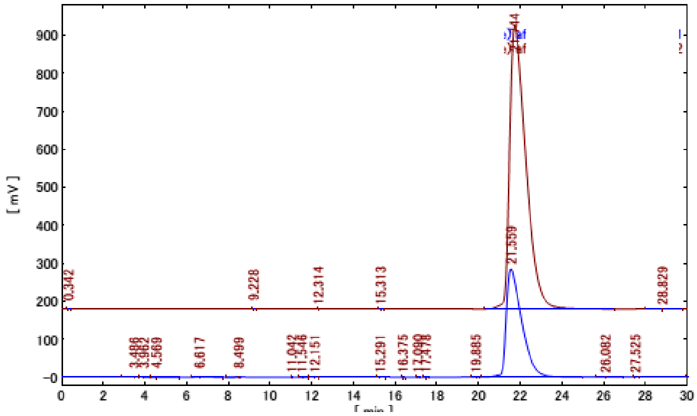

Senshu Pak PEGASIL ODS 4.6 ϕ X 250 mm

UV: 254 nm, Ex.: 366 nm, Em.: 520 nm

flow rate 1.0 mL/min with continuous gradient elution (20-50% solvent B, 0-30 min, linear) with solvent A (0.1% TFA in H_2O) and solvent B (0.1% TFA in MeCN).

Figure S29: HPLC analysis of Ir complex-peptide hybrid **4**

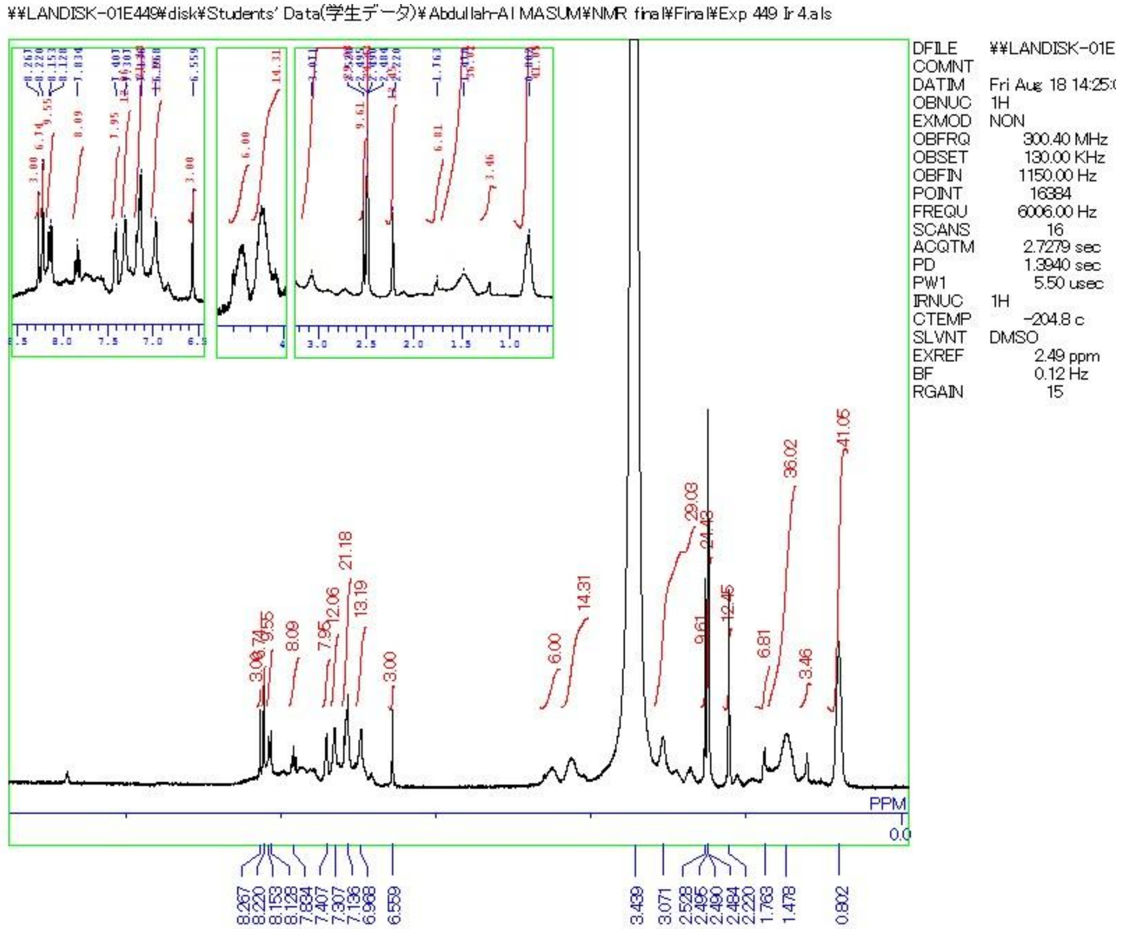

Figure S30: ^{1}H NMR chart of Ir complex-peptide hybrid **4**

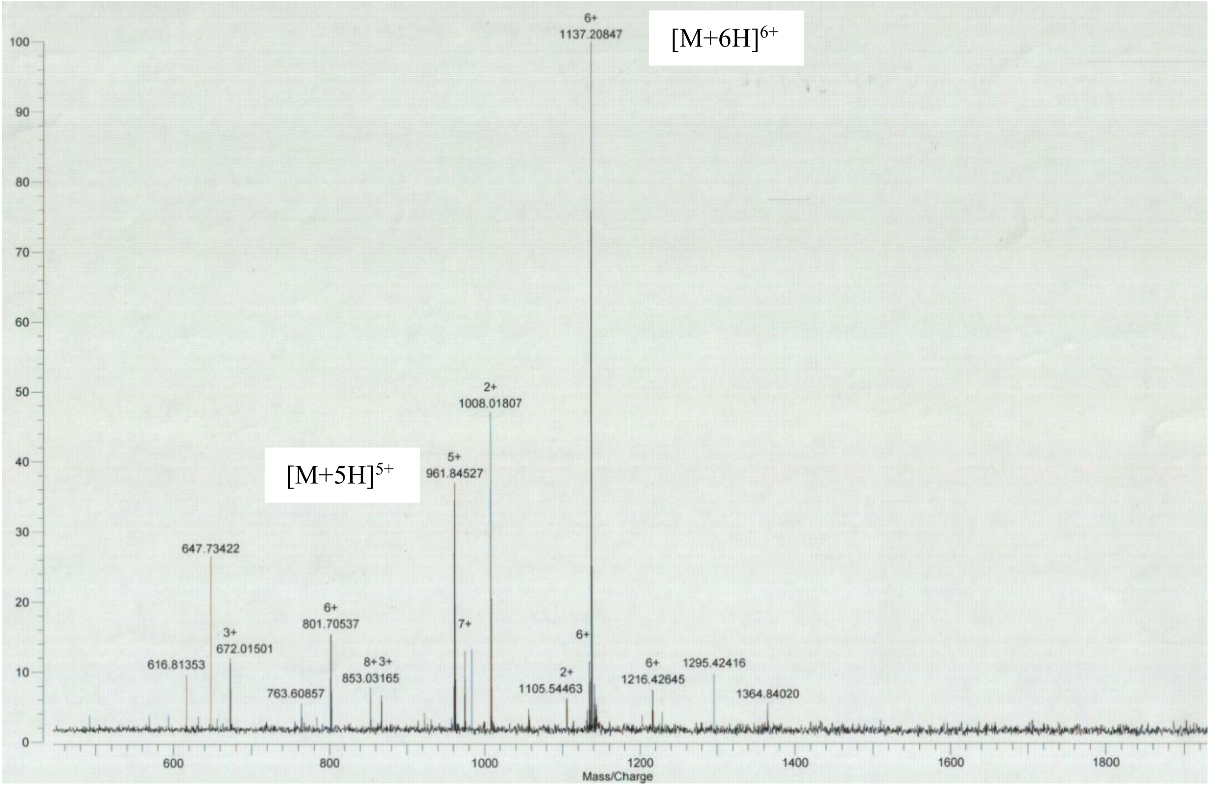

Figure S31: ESI Mass chart of Ir complex-peptide hybrid **4**

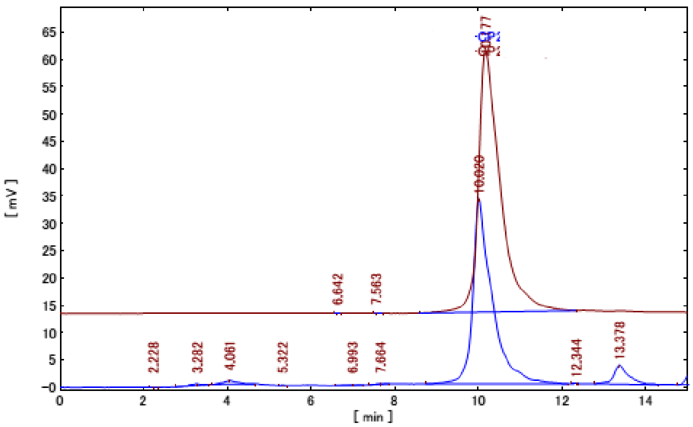

Senshu Pak PEGASIL ODS 4.6 ϕ X 250 mm

UV: 254 nm, Ex.: 366 nm, Em.: 520 nm

flow rate 1.0 mL/min with continuous gradient elution (20-50% solvent B, 0-30 min, linear) with solvent A (0.1% TFA in H_2O) and solvent B (0.1% TFA in MeCN).

Figure S32: HPLC analysis of Ir complex-peptide hybrids **5**

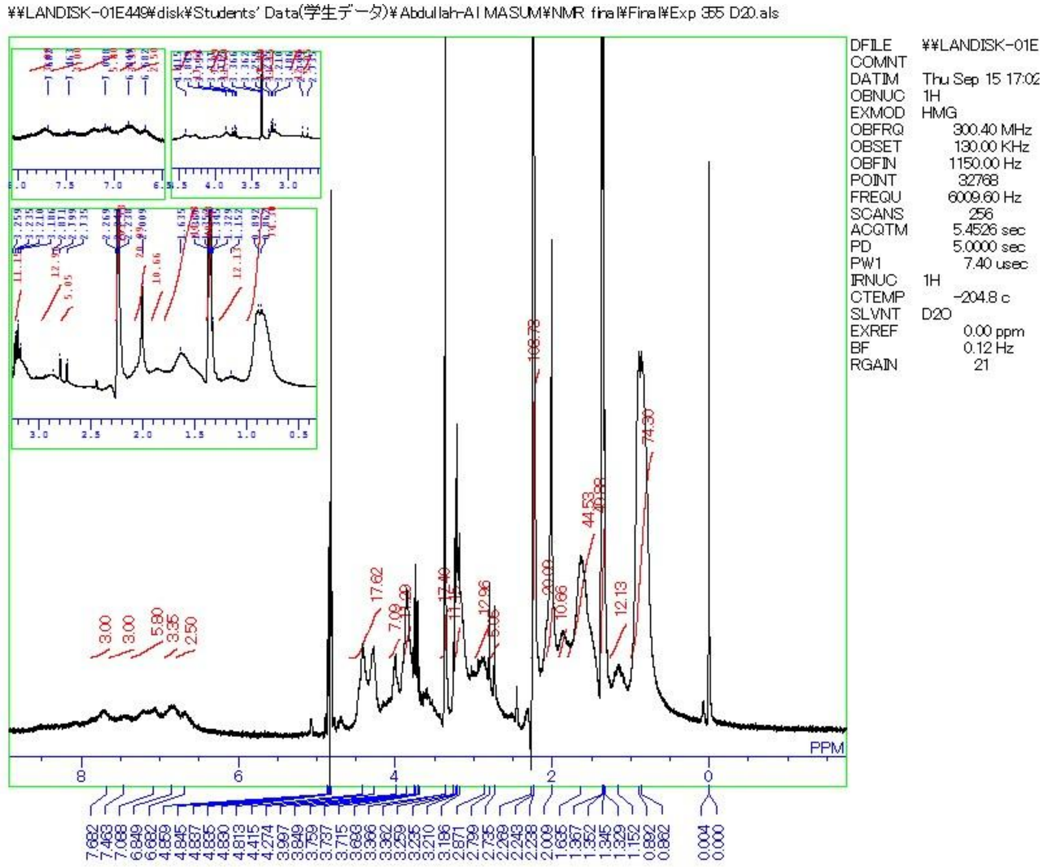

Figure S33: ^{1}H NMR chart of Ir complex-peptide hybrids **5**

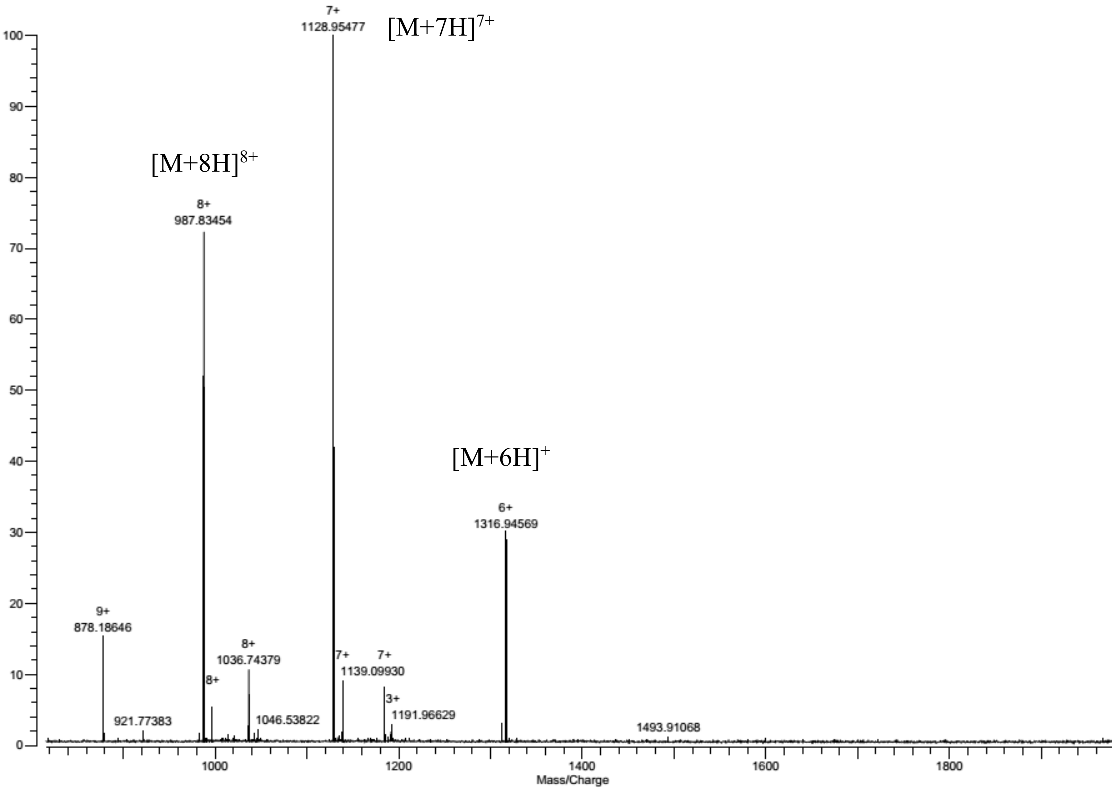

Figure S34: ESI Mass chart of Ir complex-peptide hybrid **5**

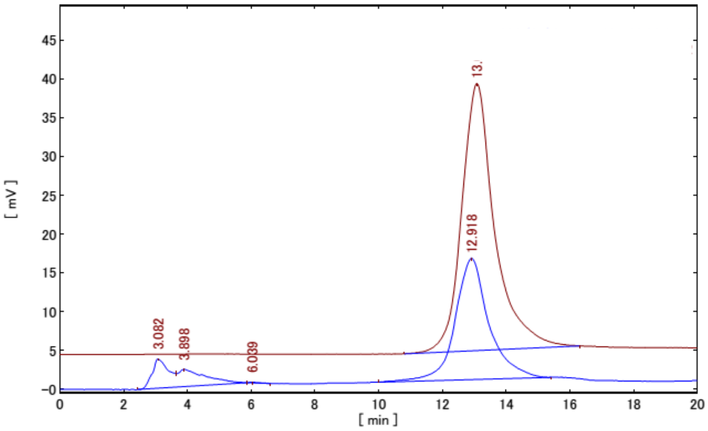

Senshu Pak PEGASIL ODS 4.6 ϕ X 250 mm

UV: 254 nm, Ex.: 366 nm, Em.: 520 nm

flow rate 1.0 mL/min with continuous gradient elution (10-40% solvent B, 0-30 min, linear) with solvent A (0.1% TFA in H_2O) and solvent B (0.1% TFA in MeCN).

Figure S35: HPLC analysis of Ir complex-peptide hybrids **6**

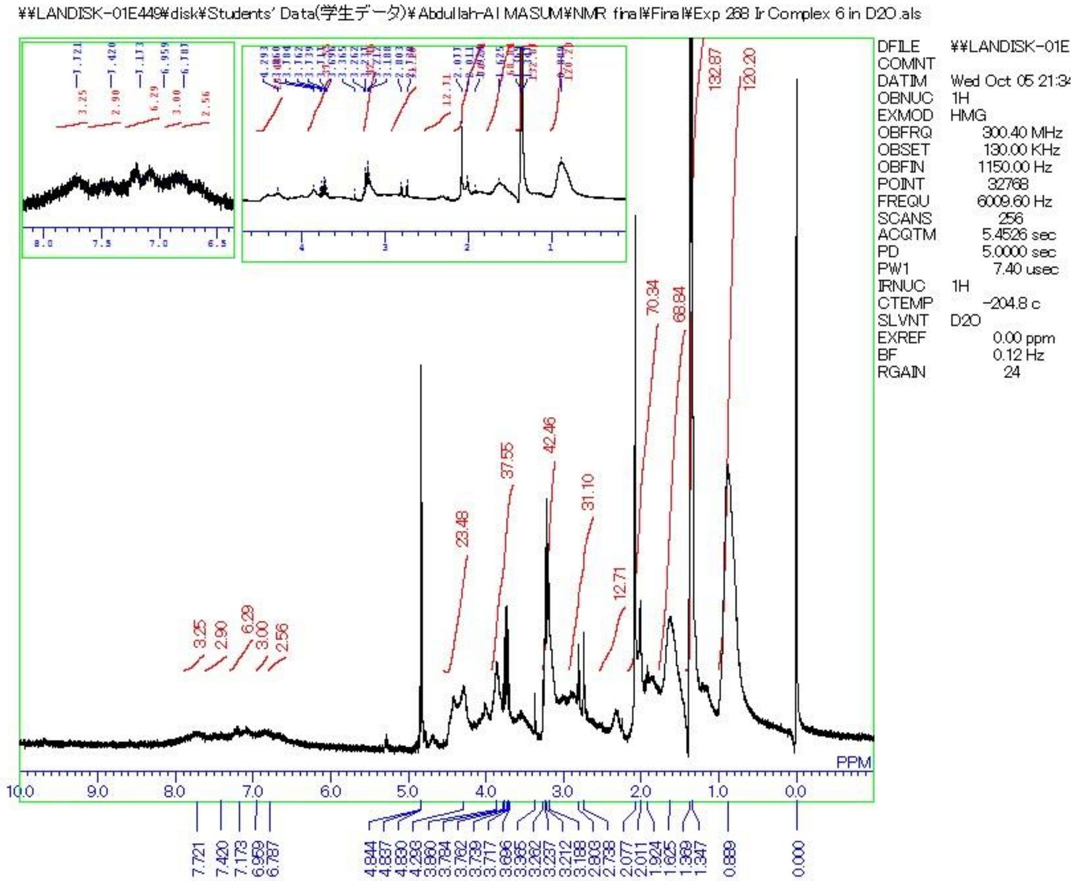

Figure S36: ^{1}H NMR chart of Ir complex-peptide hybrids **6**

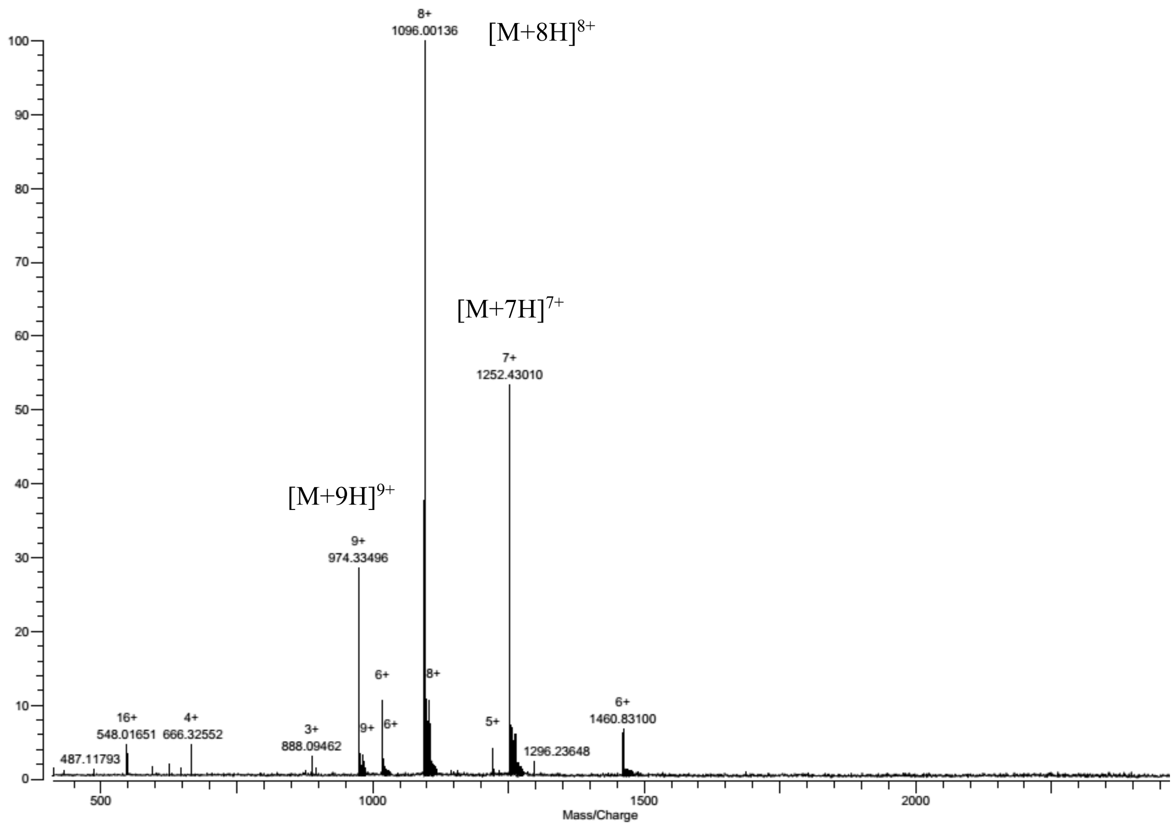

Figure S37: ESI Mass chart of Ir complex-peptide hybrid **6**
